# Adherence to Life’s Essential 8 enhances gut microbiota diversity and cognitive performance

**DOI:** 10.3389/frmbi.2025.1592023

**Published:** 2025-06-23

**Authors:** Yannick N. Wadop, Jazmyn Muhammad, Rebecca Bernal, Claudia L. Satizabal, Alexa Beiser, Ramachandran S. Vasan, Ramnik Xavier, Tiffany Kautz, Sudha Seshadri, Jayandra Jung Himali, Bernard Fongang

**Affiliations:** ^1^ Glenn Biggs Institute for Alzheimer’s and Neurodegenerative Diseases, University of Texas Health Science Center at San Antonio, San Antonio, TX, United States; ^2^ Department of Population Health Sciences, University of Texas Health Science Center at San Antonio, San Antonio, TX, United States; ^3^ Framingham Heart Study, Framingham, MA, United States; ^4^ Department of Neurology, Boston University Chobanian & Avedisian School of Medicine, Boston, MA, United States; ^5^ Department of Biostatistics, Boston University School of Public Health, Boston, MA, United States; ^6^ Department of Medicine, Section of Cardiovascular Medicine, Boston Medical Center, Boston University School of Medicine, Boston, MA, United States; ^7^ Department of Medicine, Section of Preventive Medicine and Epidemiology, Boston University School of Medicine, Boston, MA, United States; ^8^ Department of Epidemiology, Boston University School of Public Health, Boston, MA, United States; ^9^ Boston University’s Center for Computing and Data Sciences, Boston, MA, United States; ^10^ The University of Texas School of Public Health in San Antonio, San Antonio, TX, United States; ^11^ The Long School of Medicine, University of Texas Health Science Center, San Antonio, TX, United States; ^12^ Broad Institute of Massachusetts Institute of Technology and Harvard, Cambridge, MA, United States; ^13^ Center for Microbiome Informatics and Therapeutics, Massachusetts Institute of Technology, Cambridge, MA, United States; ^14^ Center for Computational and Integrative Biology and Department of Molecular Biology, Massachusetts General Hospital and Harvard Medical School, Boston, MA, United States; ^15^ Department of Medicine, University of Texas Health Science Center at San Antonio, San Antonio, TX, United States; ^16^ Department of Neurology, University of Texas Health Science Center at San Antonio, San Antonio, TX, United States; ^17^ Graduate School of Biomedical Sciences, University of Texas Health Science Center at San Antonio, San Antonio, TX, United States; ^18^ Department of Biochemistry and Structural Biology, University of Texas Health Science Center at San Antonio, San Antonio, TX, United States

**Keywords:** gut microbiota, Life’s Essential 8, cognitive performance, mediator, cardiovascular health

## Abstract

**Introduction:**

Emerging evidence suggests a complex interplay among cardiovascular health, gut microbiome composition, and cognitive function. Life’s Essential 8 (LE8), developed by the American Heart Association, includes vital metrics of cardiovascular health, such as diet, physical activity, nicotine exposure, sleep health, body mass index (BMI), blood glucose, blood lipids, and blood pressure.

**Methods:**

In this study, we analyzed data from 781 participants in the Framingham Heart Study (FHS) to explore the relationship between LE8 adherence, gut microbiota, and cognitive performance. Multivariable linear regression models and mediation analysis were used to investigate this relationship.

**Results:**

Participants with greater adherence to LE8 demonstrated significantly increased gut microbial diversity (α-diversity: Chao1, p = 0.0014; Shannon, p = 0.0071) and distinct microbial compositions (β-diversity: PERMANOVA p = 1e-4). Higher adherence to LE8 was related to an increased abundance of genera *Barnesiella* and *Ruminococcus*, while a reduced abundance of *Clostridium* was associated with higher LE8 adherence. Greater gut microbial diversity (α-diversity: Chao1, p = 0.0012; Shannon, p = 0.0066), and beneficial genera like *Oscillospira* correlated with better global cognitive scores (GCS). Taxonomic overlap analyses revealed microbial taxa that simultaneously influence both LE8 adherence and cognitive outcomes. Mediation analyses indicated that specific taxa, including *Barnesiella* and *Lentisphaerae*, mediated the link between LE8 adherence and cognitive performance. These taxa may serve as key modulators in the gut–brain axis, connecting cardiovascular and brain health. Conversely, higher *Clostridium* abundance was associated with poorer cognitive performance.

**Discussion:**

This study highlights the significance of comprehensive cardiovascular health metrics in shaping gut microbiota and enhancing cognitive resilience. Our findings underscore the therapeutic potential of targeting gut microbiota to mitigate cognitive decline, warranting further exploration through longitudinal and metagenomic studies.

## 1 Introduction

The interconnectedness of cardiovascular health, gut microbiota, and cognition has emerged as a critical focus of biomedical research. Life’s Essential 8 (LE8), established by the American Heart Association, provides a comprehensive framework to optimize cardiovascular health through key metrics: diet, physical activity, nicotine exposure, sleep health, BMI, blood glucose, blood lipids, and blood pressure (Lloyd-Jones et al., 2022). Adherence to LE8 has been associated with reduced risks of cardiovascular disease, stroke, and neurodegenerative conditions, making it an essential metric for overall health (Zhou et al., 2023; López-Bueno et al., 2024; Liu et al., 2024; Zhu et al., 2024a).

Recent studies have highlighted significant links between cardiovascular health and cognitive outcomes (Gardener et al., 2016; Kulshreshtha et al., 2019; Zimmerman et al., 2021). Poor cardiovascular health, characterized by risk factors such as hypertension, obesity, and poor sleep, has been implicated in cognitive decline and dementia (Chen et al., 2016; Walker et al., 2017; Legault et al., 2021; Mukherjee et al., 2024). Optimal adherence to LE8 metrics has been correlated with improved cognitive performance, likely due to enhanced blood flow, reduced neuroinflammation, and mitigation of vascular risk factors (De Moraes et al., 2024; Tang et al., 2024; Zhu et al., 2024a). For instance, higher adherence to LE8 has been linked to better executive function and memory performance, underscoring the importance of cardiovascular health in maintaining cognitive resilience (Dintica et al., 2024; Zhu et al., 2024a).

The gut microbiome—a complex ecosystem of trillions of microorganisms (Lozupone et al., 2012; Belizário and Faintuch, 2018)—plays a vital role in human health, influencing metabolism, immunity, and neural processes (Gomaa, 2020; Chen et al., 2021; De Vos et al., 2022; Sorboni et al., 2022). Individual components of LE8, such as diet and physical activity, are known to profoundly shape the gut microbiota (Tamayo et al., 2024). Diets rich in fiber and polyphenols promote the growth of beneficial taxa like *Faecalibacterium* and *Bifidobacterium* (Dueñas et al., 2015; Liu et al., 2019; Loo et al., 2020; Rodríguez-Daza et al., 2021), while physical activity has been shown to enhance microbial diversity and abundance of short-chain fatty acid (SCFA)-producing bacteria (Campbell and Wisniewski, 2017; Codella et al., 2018; Aya et al., 2021; Pérez-Prieto et al., 2024). Conversely, suboptimal adherence to LE8, such as diets high in saturated fats and sedentary lifestyles, promotes dysbiosis, characterized by reduced microbial diversity and an overrepresentation of pathogenic taxa (Malesza et al., 2021; Martinez et al., 2021; Thomas et al., 2022). This dysbiosis is increasingly recognized as a precursor to systemic inflammation and chronic disease (Logan et al., 2016; Furman et al., 2019).

The gut–brain axis provides a mechanistic link between the gut microbiome and cognitive function (Morais et al., 2021). Microbial metabolites, such as SCFAs, play neuroprotective roles by reducing inflammation, enhancing the integrity of the blood–brain barrier, and modulating neurotransmitter synthesis (Silva et al., 2020; Mirzaei et al., 2021; O’Riordan et al., 2022). Dysbiosis has been implicated in cognitive impairment through mechanisms involving systemic inflammation, oxidative stress, and altered neurochemical signaling (Liu et al., 2020; Shandilya et al., 2022). Emerging research suggests that the gut microbiome may mediate the association between LE8 adherence and cognitive outcomes. For example, individuals adhering to healthy lifestyle practices (measured by LE8) exhibit microbiomes enriched with taxa associated with neuroprotection, such as *Barnesiella* and *Ruminococcus* (Tamayo et al., 2024). These taxa are thought to modulate brain function via SCFA production and anti-inflammatory pathways.

Despite these associations, the interplay between LE8, the gut microbiome, and cognition remains underexplored. Understanding whether and how the gut microbiome mediates the LE8-cognition link could reveal novel therapeutic targets and inform strategies for optimizing both cardiovascular and brain health. This study aims to elucidate these relationships, hypothesizing that higher adherence to LE8 is associated with a more diverse and balanced gut microbiome, which mediates its protective effects on cognition.

## 2 Materials and methods

### 2.1 Study design and participants

This study included participants from the Framingham Heart Study (FHS) who had available data on LE8 metrics, gut microbiome sequencing, and cognitive assessments. The FHS is a longitudinal community-based study initiated in 1948, with multiple offspring cohorts enrolled in subsequent decades (Andersson et al., 2019; Fongang et al., 2023). Participants in our analysis were drawn from the 3^rd^ Generation cohort, New Offspring Spouses cohort, and the minority oversample Omni Group 2, who attended a clinic examination that included stool sample collection for microbiome analysis and comprehensive clinical evaluation (2016–2019). Eligible individuals were adults who provided a stool sample and underwent cognitive testing in approximate temporal proximity. All participants gave written informed consent, and the study protocol was approved by the Institutional Review Board of Boston University.

### 2.2 Life’s Essential 8 score assessment

LE8 scores for each participant were computed according to the American Heart Association (AHA) guidelines for LE8, with minor adaptations for available FHS data (Lloyd-Jones et al., 2022; Carbonneau et al., 2023). Each of the eight health domains (diet, physical activity, nicotine exposure, sleep health, BMI, blood glucose, blood lipids, blood pressure) was calculated and scored on a scale from 0 to 100, with higher scores indicating better (healthier) status in that domain (Kannel and Sorlie, 1979; Mellen et al., 2008). Briefly, diet score was calculated using Dietary Approaches to Stop Hypertension (DASH) based on components including the intake of vegetables, fruits, nuts, legumes, and whole grains; as well as low-fat dairy, and the intake of red and processed meat, sugar-sweetened beverages, and sodium (Kannel and Sorlie, 1979; Mellen et al., 2008). The physical activity score was computed by considering the duration and strength of activities, including sleep, sedentary, and different strengths of activities (i.e., light, moderate, and vigorous) (Kannel and Sorlie, 1979). The other scores, such as sleep quality, blood sugar levels, BMI, blood lipids, and blood pressure, were derived based on the standard of the AHA (Lloyd-Jones et al., 2022). The overall LE8 score was estimated as the unweighted average of the eight component scores, also yielding a composite range from 0 to 100, with higher scores reflecting overall better cardiovascular health. In our cohort, the LE8 score distribution was approximately normal, with a mean of 76.3. Based on AHA-established cutoffs (Lloyd-Jones et al., 2022), participants were initially categorized into three adherence levels: low (score 0–49), moderate (50–79), and high (80–100). These thresholds align with AHA guidelines for poor, intermediate, and ideal cardiovascular health metrics. In our study sample, very few individuals (n = 13) fell into the low adherence category (LE8< 50). Therefore, for analytical purposes, we combined the low and moderate groups into a single category (“ModLow”) to ensure adequate group sizes. Thus, participants were finally classified into two categories: High LE8 adherence (score 80–100) and ModLow LE8 adherence (score< 80).

To provide insight into the composition of the LE8 score, we also examined how each individual component correlated with the overall score. We performed a Spearman correlation analysis between each of the eight component scores and the total LE8 score across participants. All eight components showed a positive correlation with the overall LE8 score (each correlation p-value< 0.05), indicating that improvements in any single domain contributed to a higher composite score (with correlation coefficients ranging from ρ ≈ 0.2 to 0.6) as displayed in **Supplementary Figure S1**. This supports the interpretation of the LE8 score as an integrated measure of cardiovascular health.

### 2.3 Gut microbiome collection and analysis

Stool samples were collected from FHS participants following standardized procedures to ensure sample integrity, as previously described (Walker et al., 2021; Fongang et al., 2023; Okoro et al., 2023). Briefly, participants were provided stool collection kits and instructed on proper sample collection. Samples were immediately frozen and stored at –80°C until analysis. Microbial DNA was extracted using the Qiagen PowerSoil DNA Isolation Kit (Qiagen, Hilden, Germany) according to the manufacturer’s protocol. The V4 region of the 16S rRNA gene was amplified from each sample and sequenced on the Illumina MiSeq platform, generating paired-end reads (2 × 250 bp). Sequence data were processed using the DADA2 pipeline (Okoro et al., 2023) to infer Amplicon Sequence Variants (ASVs), followed by taxonomic assignment against the SILVA reference database (version 138). The processing steps included quality filtering, denoising, chimera removal, and merging of paired reads, resulting in high-quality sequences for analysis.

Across the 781 samples, sequencing depth was high: each sample yielded on the order of tens of thousands of reads (median approximately ~18,000 reads per sample). After DADA2 processing and filtering, we identified a rich microbial community comprising over 200 distinct genera, spanning 12 bacterial phyla in total. To focus on prevalent taxa and reduce sparsity, we excluded very rare ASVs/features; in particular, features not present in at least 10% of samples at a relative abundance of ≥0.1% were filtered out (this threshold aligns with the prevalence cutoff used in downstream association analyses). The remaining microbiome data included predominantly gut-associated phyla such as *Firmicutes*, *Bacteroidetes, Actinobacteria, Proteobacteria, Verrucomicrobi*a, and others, and hundreds of genera, providing a robust foundation for diversity and association analyses.

### 2.4 Global Cognitive Score assessment

To assess cognitive performance, participants completed a neuropsychological test battery administered by trained examiners during their FHS clinic visit, as previously described (Au et al., 2004). The current study included the following tests: Trail Making Test Part B (a measure of executive function and processing speed), Logical Memory (immediate and delayed recall, assessing verbal memory), Visual Reproduction (immediate and delayed recall, assessing visual memory), and Similarities (a subtest of the Wechsler Adult Intelligence Scale, measuring verbal abstract reasoning and executive function) (Elwood, 1991; Drozdick et al., 2018). Trail Making B times were log-transformed to normalize the distribution, and the sign was inverted so that higher values on all tests uniformly indicated better performance. We then constructed a Global Cognitive Score (GCS) by applying principal component analysis (PCA) to the battery of test scores (with each test standardized). The PCA yielded a first principal component (PC1) that accounted for the largest share of variance across the tests. This single component explained more than 42% of the total variance in the cognitive test battery, consistent with prior studies that have derived a general cognitive factor in community-based cohorts (e.g., ARIC, CHS, FHS offspring) (Pase et al., 2016; Pase et al., 2023; Yiallourou et al., 2024; Young et al., 2024). We used PC1 as the summary measure of global cognition for each participant; higher GCS values indicate better overall cognitive performance. By construction, the GCS is a weighted linear combination of the individual test scores (with loadings approximately reflecting each test’s correlation with the general factor). Thus, GCS captures broad cognitive function, combining elements of memory, executive function, and processing speed into a single metric. This GCS was treated as a continuous outcome in primary analyses. For secondary analyses (e.g., comparing microbiome features between cognitively “normal” vs. “poor” performers), we dichotomized the GCS: participants in the lowest quintile of GCS (bottom 20%) were classified as having “poor” cognitive performance, and those in the upper 80% were classified as “normal.” This grouping was used to illustrate differences in microbiome diversity and composition between individuals with relatively low vs. higher cognitive function.

### 2.5 Study context, hypotheses, and objectives

**Figure 1:**
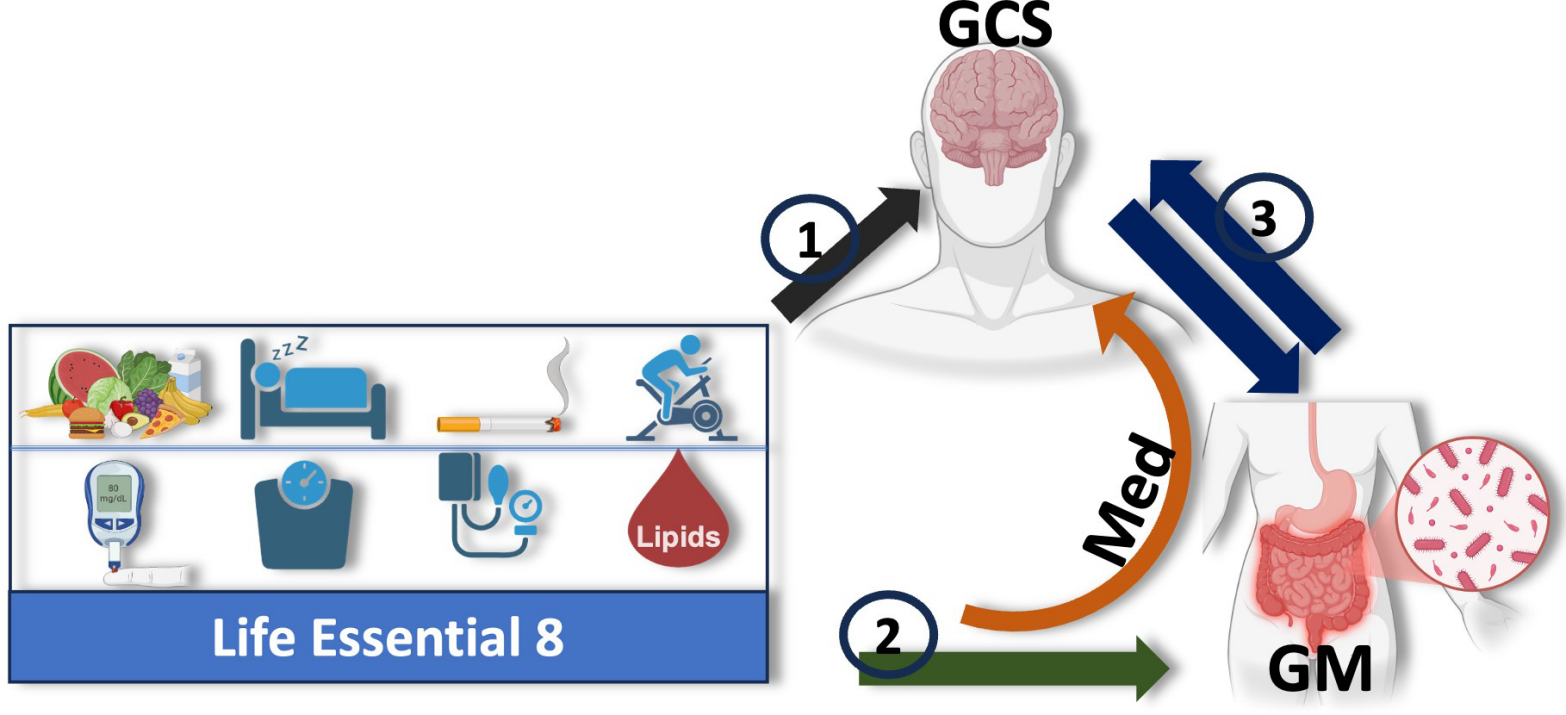
Study design: Illustration of the hypothesized mediating role of the gut microbiome (GM) in the relationship between adherence to Life’s Essential 8 (LE8) and cognition, measured by the Global Cognitive Score (GCS). The study is structured around three key objectives: (1) assess the relationship between overall LE8 adherence, its individual components, and GCS; (2) evaluate how LE8 adherence and its components influence gut microbiome composition and diversity; and (3) explore bidirectional interactions between the gut microbiome and cognition. The gut microbiome is proposed to mediate the association between LE8 adherence and GCS, with specific microbial taxa or pathways potentially driving this relationship. The orange arrow highlights the central mediating role of the gut microbiota in the LE8–cognition link.

**Figure 1** illustrates our study framework, investigating how cardiovascular health (LE8 adherence) relates to cognitive performance and the potential mediating role of the gut microbiome. Our research addresses three key objectives: (1) LE8 and cognition: test the association between overall LE8 adherence (and its individual components) and global cognitive performance (GCS); (2) LE8 and gut microbiome: evaluate the relationship between LE8 adherence (overall and component-wise) and gut microbiome composition (diversity and taxa abundance); and (3) Gut microbiome and cognition: assess the associations between gut microbiome features and cognitive performance, and (4) specifically test whether the microbiome mediates the LE8–cognition association. We hypothesize that higher LE8 adherence will correlate with better cognition, and that a more favorable gut microbiome (greater diversity, higher abundance of beneficial taxa) will be associated with both high LE8 adherence and better cognition. Further, we hypothesize that some of the effect of LE8 on cognition is transmitted via changes in the gut microbiome, i.e., specific microbial taxa will show mediation effects in the LE8–cognition pathway.

### 2.6 Statistical analysis

#### 2.6.1 Participant characteristics and descriptive analyses

We first compared baseline characteristics between the High and ModLow LE8 adherence groups. Continuous variables were summarized with means (± standard deviation) or medians [quartiles] and compared using t-tests or Wilcoxon rank-sum tests as appropriate. Categorical variables were summarized as counts (percentages) and compared using χ² tests. Key variables included age, sex, education, race, vascular risk factors, and prevalence of cardiovascular disease and diabetes. This provided context on whether the two LE8 groups differed on factors that might also relate to microbiome or cognition.

#### 2.6.2 Multivariable association analysis

To evaluate associations between LE8 adherence, gut microbiome features, and cognitive performance, we used multivariable linear regression implemented through the MaAsLin2 R package (version 1.8.0). MaAsLin2 (Microbiome Multivariable Association with Linear Models) is a pipeline designed to find associations between microbial abundances and metadata while adjusting for covariates (Mallick et al., 2021). For our analyses, we set MaAsLin2 parameters to ensure rigorous control of the data: we applied cumulative sum scaling (CSS) normalization to raw count data to account for varying sequencing depth across samples, and we specified no additional transformation of the normalized data (setting transform = “NONE”). We used a negative binomial regression model for association testing (suitable for overdispersed count or relative abundance data). We set the minimum feature abundance threshold to 1×10^−3^ (0.1%) and required a minimum prevalence of 10% of samples for a feature to be analyzed. We adjusted p-values for multiple testing using the Benjamini–Hochberg false discovery rate (FDR) method, considering q< 0.05 as significant. All models included relevant covariates as described below.

We performed two primary sets of association analyses using MaAsLin2: (a) associations between gut microbial taxa (at various taxonomic levels) and LE8 adherence; and (b) associations between gut microbial taxa and cognitive performance (GCS).

For case (a), the model took the form:


Taxon_abundance=LE8_score+Age+Sex

where *Taxon_abundance* is the normalized count (relative abundance) of a given bacterial taxon (e.g., a genus) and LE8_score is the continuous overall LE8 score. Covariates included age and sex (we did not include education or time difference here because LE8 adherence is unlikely to be causally affected by those, and we wanted to maximize power to detect microbiome associations with LE8). This model yielded β coefficients representing the change in taxon abundance per unit increase in LE8 score (with 95% confidence intervals and p-values). We also ran complementary models using LE8 adherence groups (High vs. ModLow) instead of the continuous score, which gave very similar results (differences largely in effect scaling).

For case (b), the association model was:


$$GCS=Taxon\_abundance+Age+Age^{2}+BMI+Sex+\;Education+time\_interval$$

where GCS is the global cognitive score and the covariates included age, sex, body mass index (BMI), education (highest attained degree), and *time_interval* represents the time difference between stool collection and cognitive tests between stool collection and cognitive testing (in weeks). These covariates were selected based on known associations with cognitive function or microbiome composition. This model’s β coefficients represent the change in taxon abundance per unit increase in cognitive score. We also examined an alternative dichotomous cognition outcome (poor vs. normal, as defined earlier) using logistic regression for select analyses, but the primary reported results treat GCS as continuous.

#### 2.6.3 Microbiome diversity analysis

We assessed alpha-diversity and beta-diversity to compare overall microbial community structure between groups. Alpha-diversity was quantified using the Chao1 and Shannon diversity indexes for richness and evenness (computed using the R package *MicrobiotaProcess*) (Xu et al., 2025). We compared alpha-diversity between High vs. ModLow LE8 adherence groups using the Wilcoxon rank-sum test (non-parametric). Similarly, we compared alpha-diversity between Normal vs. Poor cognition groups (GCS top 80% vs. bottom 20%) with Wilcoxon tests. For continuous associations, we fit multivariable linear regression models with alpha-diversity as the outcome and LE8 score or GCS as the predictor (adjusting for the same covariates as in the taxa models above). These models were used to confirm whether higher LE8 score predicted higher diversity (and whether higher GCS predicted higher diversity) while controlling for confounders such as age and sex (and additionally BMI, education, time interval in GCS models).

Beta-diversity (between-sample diversity) was evaluated using the Bray–Curtis dissimilarity. We visualized beta-diversity via Principal Coordinates Analysis (PCoA) plots. To test differences in overall microbiome composition between groups (High vs. ModLow LE8; Normal vs. Poor cognition), we performed permutational multivariate analysis of variance (PERMANOVA) using the adonis2 function from the vegan R package (Dixon, 2003). Each PERMANOVA was run with 1000 permutations. We conducted PERMANOVA both without covariate adjustment and with covariates included as conditioning variables. For LE8 groups, we adjusted for age and sex; for cognition groups, we adjusted for age, sex, BMI, education, and time interval. This approach allowed us to determine if group differences in microbiome composition remained significant after accounting for these factors. The PERMANOVA results are reported as p-values for the overall group effect on microbiome community structure.

#### 2.6.4 Mediation analysis

To explore whether the gut microbiome mediates the association between LE8 adherence and cognitive performance, we performed a causal mediation analysis using the R package mediation (Tingley et al., 2014). From the association analyses, we identified candidate mediator taxa as those that showed significant associations with *both* LE8 (predictor) and GCS (outcome) in the models described above. For each candidate taxon, we set up two regression models consistent with a mediation framework:

- Mediator model: *Taxon_abundance ~ LE8_adherence + age + sex*
- Outcome model: *GCS ~ LE8_adherence + Taxon_abundance + age + sex + education*

We treated LE8 adherence as continuous (LE8 score) in these models. Taxon abundances were handled as in MaAsLin2 (CSS-normalized counts). We used generalized linear models for both mediator and outcome models.

The fitted objects were used as inputs in the *mediate()* function of the mediation package (Tingley et al., 2014) to estimate the indirect effect of LE8 on GCS via the taxon (the mediated effect). We performed non-parametric bootstrap resampling (1000 simulations) to compute confidence intervals and p-values for the mediated effect. An indirect (mediated) effect was considered statistically significant if the bootstrap p< 0.05. We reported the mediation effect size as “indirect effect (IDE)” along with its 95% CI and p-value.

## 3 Results

### 3.1 Participant characteristics

The study cohort consisted of 781 participants (57.1% female) aged 32 to 78 years (mean age 54.9 ± 8.3 years). The majority (~64.4%) had a college-level education or higher. The overall LE8 score ranged from 27.5 to 100, with a mean of 76.3 (indicating generally intermediate-to-high cardiovascular health). Based on the LE8 score, 322 individuals (~41%) were classified as High LE8 adherence (score 80–100), and 459 (~59%) as ModLow LE8 adherence (score< 80, combining moderate and low categories, though only 13 were truly low). By design, the High adherence group had significantly better profiles on all individual LE8 components (each component score was higher on average, p< 0.001 for all). Participants in the high LE8 group also had a slightly lower mean age than those in the ModLow group (53.2 vs. 56.2 years, p = 1×10^−6^). Prevalence of hypertension treatment, diabetes, and cardiovascular disease was lower in the High adherence group, consistent with their healthier profiles (for example, diabetes: 1.7% vs. 98.3%, p = 2.2×10^−16^). Importantly, GCS was higher among participants with high LE8 adherence compared to those with moderate/low adherence. In unadjusted comparison, the High group’s median GCS was significantly greater than the ModLow group’s (0.71 in High vs. 0.47 in ModLow, p = 2.2×10^−6^), indicating better cognitive performance in the high cardiovascular health group. **Table 1** summarizes the key demographic and health characteristics by LE8 group.

**Table 1:** Demographic characteristics.

Variables	Overall (N = 781)	High LE8 (N = 322)	ModLow (N = 459)	P-value
Age, years	54.9 ± 8.3	53.2 ± 8.5	56.2 ± 7.9	1.00e-6
Female, n (%)	446 (57.1)	227 (70.5)	219 (47.7)	0.64
Education, n (%)				
High school degree	76 (9.7)	15 (4.7)	61 (13.3)	2.88e-13
Some college	202 (25.9)	57 (17.7)	145 (31.6)	2.20e-16
College graduated	503 (64.4)	250 (77.6)	253 (55.1)	0.89
Time interval between stool collection and cognitive test, weeks	133.9 ± 124.2	131.2 ± 120.5	135.9 ± 126.8	0.59
Cardiovascular risk factors				
Triglycerides, mg/dL	110.9 ± 78.3	77.6 ± 32.2	132.9 ± 92.5	2.00e-16
Systolic blood pressure, mmHg	119 ± 14	113 ± 12	123 ± 14	2.00e-16
Diastolic blood pressure, mmHg	75 ± 8	72 ± 7	78 ± 9	2.00e-16
Total cholesterol, mg/dL	190.1 ± 35.0	185.1 ± 31.1	193.6 ± 37.1	5.16e-4
HDL cholesterol, mg/dL	61.9 ± 19.8	69.5 ± 19.2	56.5 ± 18.4	2.00e-16
Treatment for hypertension, n (%)	179 (22.9)	24 (13.4)	155 (86.6)	2.20e-16
Stage I hypertension, n (%)	221 (28.3)	32 (14.5)	189 (85.5)	2.20e-16
Prevalent of cardiovascular disease, n (%)	30 (3.8)	10 (33.3)	20 (66.7)	2.01e-2
Current smoking, n (%)	32 (4.1)	1 (3.1)	31 (96.9)	4.17e-13
History of Diabetes, n (%)	58 (7.4)	1 (1.7)	57 (98.3)	2.20e-16
Body mass index, kg/m^2^, median [Q1, Q3]	26.8 [24.1, 30.6]	24.2 [22.1, 26.3]	29.5 [26.4, 32.8]	2.20e-16
Global cognition	0.59 [0.12, 1.09]	0.71 [0.28, 1.18]	0.47 [0.01, 0.45]	2.16e-6
Trail Making part B	0.07 [−0.17, 0.31]	0.11 [−0.09, 0.35]	0.03 [−0.19, 0.24]	5.00e-5
Similarities	17.88 ± 2.89	18.05 ± 2.79	17.77 ± 2.97	0.19
Visual Reproduction	19.04 ± 4.72	19.89 ± 4.38	18.44 ± 4.85	1.60e-5
Logical Memory	24.79 ± 6.66	25.71 ± 6.92	24.15 ± 6.41	1.50e-3
Life’s essential eight scores	76.3 ± 11.9	87.5 ± 5.2	68.4 ± 8.4	2.00e-16
Diet scores	45.2 ± 30.2	62.3 ± 27.7	33.2 ± 25.8	2.00e-16
Sleep health scores	89.9 ± 18.2	94.7 ± 12.2	86.6 ± 20.8	1.70e-11
Nicotine exposure scores	87.4 ± 22.3	94.3 ± 11.9	82.5 ± 26.3	2.00e-16
Physical activity scores	83.2 ± 27.9	90.9 ± 16.9	77.8 ± 32.6	6.40e-13
Blood pressure scores	72.3 ± 28.4	87.3 ± 20.4	61.8 ± 28.5	2.00e-16
Blood glucose scores	92.3 ± 19.4	98.3 ± 8.3	88.1 ± 23.4	2.00e-16
Blood lipids scores	72.9 ± 26.9	85.5 ± 21.1	64.0 ± 27.2	2.00e-16
Body mass index scores	66.9 ± 30.6	86.8 ± 18.5	53.0 ± 29.8	2.00e-16

Values are mean±SD, or median [quartile 1, quartile 3] for nonnormally distributed variables. ModLow indicates LE8 group combining individuals with low and moderate LE8 adherence. The tests of difference between both LE8 groups were based on t-test for continuous variables or test of proportion for categorical variables.

In terms of gut microbiome data, as noted in Methods, sequencing coverage was robust for all individuals. No sample had fewer than 5,000 reads; the median sequencing depth was around 18k reads per sample (Q1–Q3: ~12k–25k). A total of ~4,500 ASVs were inferred across all samples, clustering into >200 genera across 12 phyla. The most abundant bacterial phyla overall were *Firmicutes* and *Bacteroidetes*, followed by *Actinobacteria* and *Proteobacteria* (with smaller contributions from *Verrucomicrobia*, *Fusobacteria*, *Lentisphaerae*, etc.). The median Shannon diversity index was 3.7 (inter quartile range 3.3–4.1), indicating a fairly diverse gut microbiome on average. These descriptive statistics provide a context that our cohort’s microbiome diversity and composition are comparable to other adult populations. We next report the associations of LE8 adherence with gut microbiome features and subsequently their links to cognitive outcomes.

### 3.2 Adherence to LE8 is associated with increased gut microbial diversity

**Figure 2:**
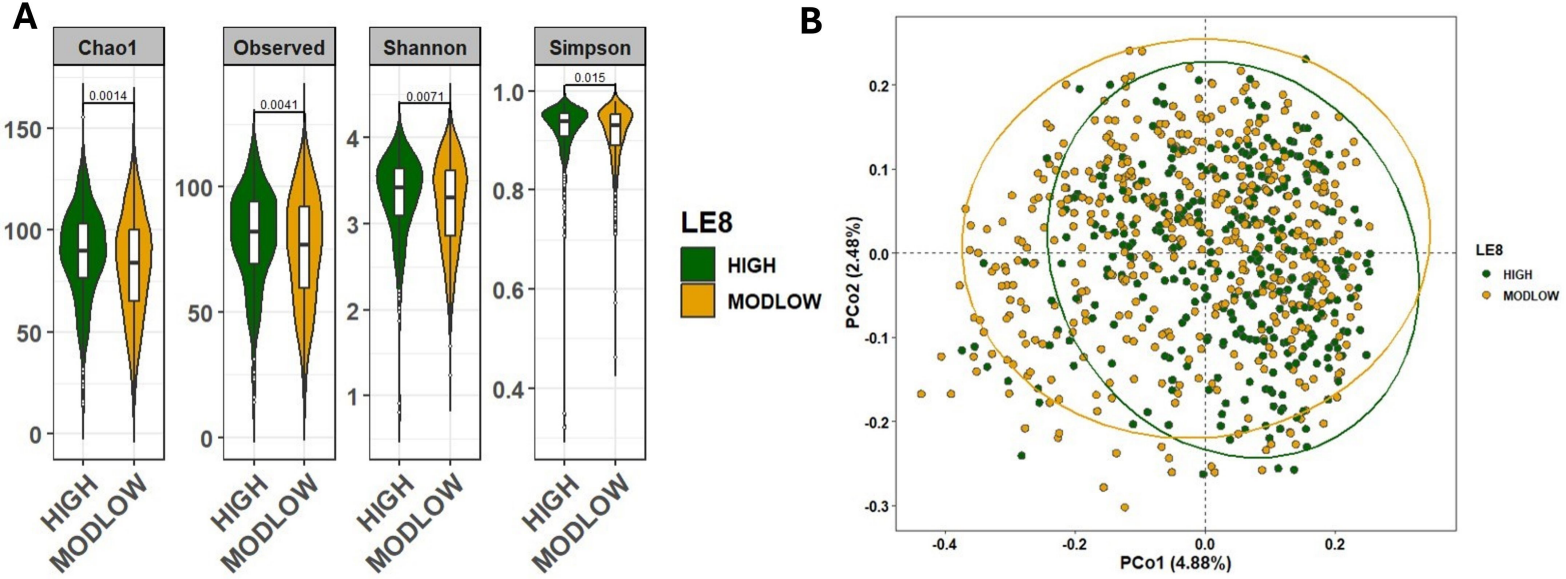
Comparison of the microbial community diversity of samples between high and below high (ModLow) LE8 adherence groups. **(A)** The α-diversity analysis through calculation of Chao1, Observe, Shannon, and Simpson indexes. The tests of difference in microbial diversity at the OTU level between both LE8 groups were based on Wilcoxon test. **(B)** Beta diversity on samples represented by PCoA using Bray–Curtis dissimilarity. The p-value of PERMANOVA test is 1.0e-4.

We first examined whether overall gut microbiome diversity differed by LE8 adherence. Alpha-diversity indices were significantly higher in the High LE8 adherence group compared to the ModLow group (**Figure 2A**). Specifically, the median Chao1 richness in the High group was significantly greater than in the ModLow group (Wilcoxon rank-sum p = 0.0014). Similarly, the Shannon diversity index was higher in the High LE8 group (median 3.9 vs. 3.6; p = 0.0071). These results indicate that individuals with better cardiovascular health (as captured by LE8) tend to harbor richer and even microbial communities in their gut. To ensure that these differences were not confounded by age or sex, we also performed a multivariable linear regression of each diversity measure on continuous LE8 score, adjusting for age and sex. The regression confirmed the association: each 10-point increase in LE8 score was associated with an estimated increase of +0.35 in Shannon index (β = 0.0345 per point, 95% CI 0.0208–0.0482; p = 9.88×10^–7^) and +4.0 in Chao1 (β = 0.404 per point, 95% CI 0.303–0.505; p = 3.14×10^–9^). Thus, both categorical and continuous analyses consistently show that higher LE8 adherence correlates with greater gut microbiome diversity.

We next assessed beta-diversity, which reflects differences in overall microbial community composition between groups. **Figure 2B** displays PCoA plots of Bray–Curtis dissimilarity, illustrating some separation between the two LE8 groups. Using PERMANOVA, we found that the High vs. ModLow LE8 groups have significantly distinct microbiome profiles (p = 1×10^−4^). This indicates that, considering the microbiome as a whole (all taxa abundance profiles), there is a detectable difference associated with cardiovascular health status. When we adjusted for age and sex in the PERMANOVA (essentially comparing residual microbiome differences after accounting for those factors), the group difference remained significant (PERMANOVA p = 1×10^−3^). These results suggest that the composition of the gut microbiome varies with LE8 adherence beyond what would be expected by chance or due to basic demographic differences. Key phylum-level differences included a trend for the High adherence group to have relatively more *Firmicutes* and *Lentisphaerae* and slightly less *Proteobacteria*, although overall, *Firmicutes* and *Bacteroidetes* dominated both groups (**Supplementary Figure S2**). At the family and genus levels, some taxa were noticeably more abundant in the High group, such as *Ruminococcaceae* and *Faecalibacterium* (a well-known beneficial genus in the *Ruminococcaceae* family), whereas certain taxa like *Enterobacteriaceae* (family containing some opportunistic pathogens) appeared more represented in the ModLow group (**Supplementary Figures S2, S3**).

As a sensitivity analysis, we repeated the diversity comparisons after excluding the few individuals with very low LE8 scores (< 50). This left a comparison of High vs. moderate adherence only. The results were consistent: alpha-diversity remained significantly higher in the High vs. moderate group (Chao1 p = 0.0078; Shannon p = 0.024; **Supplementary Figure S4A**), and beta-diversity differences were still significant (PERMANOVA p = 1×10^−4^; **Supplementary Figure S4B**). Thus, even when considering a more stringent contrast, better cardiovascular health was linked to a more diverse and distinct gut microbiome.

### 3.3 Adherence to LE8 is associated with specific patterns of gut microbial abundance

**Figure 3:**
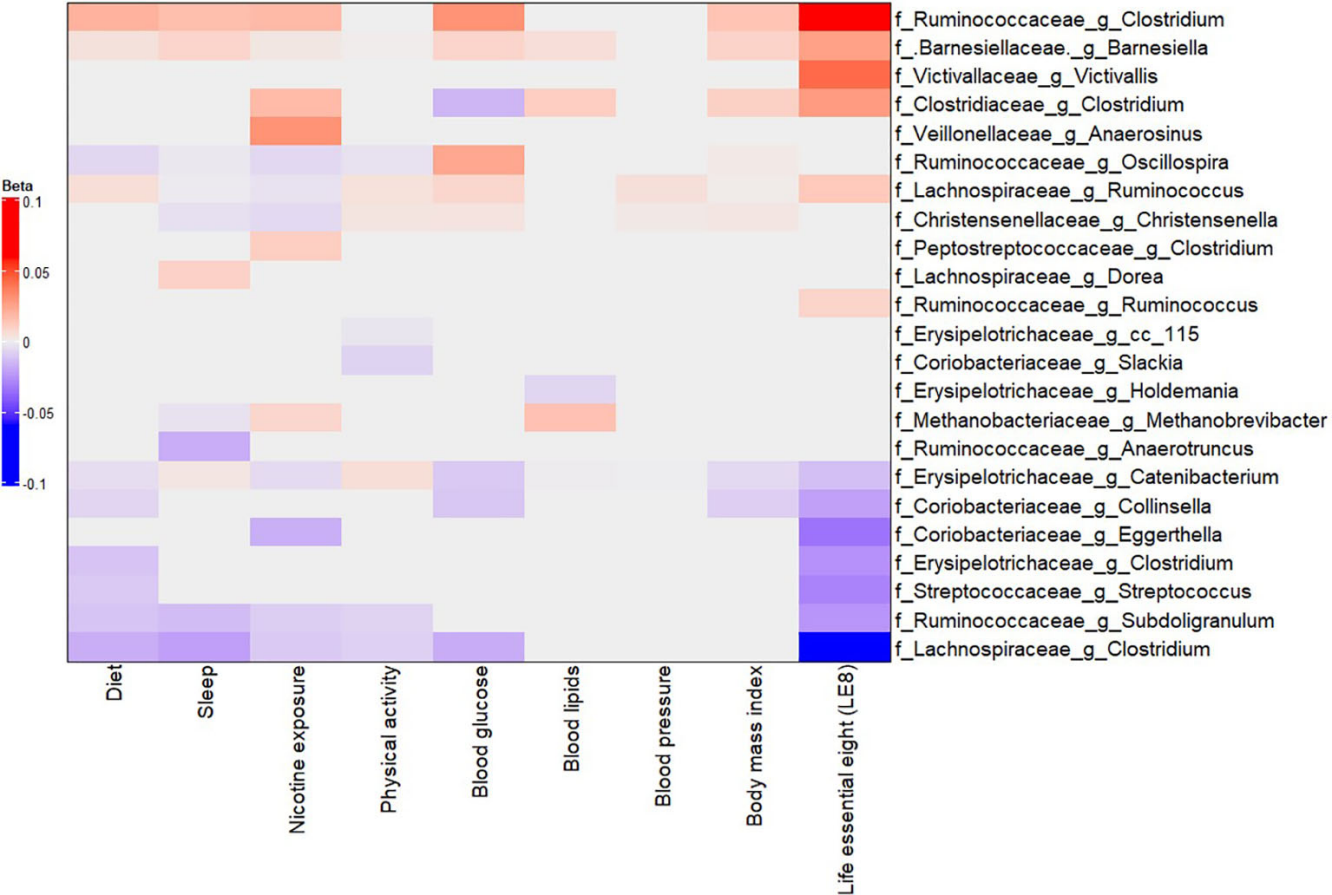
Multivariable association between gut microbiota and LE8 adherence. Heatmap depicting the significant (adjusted p-value< 0.05) genera associated with LE8 adherence along with its components.

We next examined the associations between LE8 adherence and the relative abundances of specific gut microbes. Using multivariable linear models in MaAsLin2, we regressed each taxon’s abundance on the continuous LE8 score, adjusting for age and sex. We considered results significant at FDR q< 0.05. **Figure 3** (and **Supplementary Figure S5**) presents heatmaps of the significant associations at phylum, family, and genus levels.

Our analysis identified several taxa whose abundance was significantly associated with LE8 scores. Higher LE8 scores (better cardiovascular health) showed positive associations with the abundance of several beneficial or commensal genera, including: *Barnesiellaceae Barnesiella*: β = 0.0263 (95% CI 0.0261–0.0266), p = 1.4×10^−297^. This indicates that individuals with higher LE8 scores tend to have a higher relative abundance of *Barnesiella*. The extremely low p-value reflects both a consistent direction and relatively small variance due to the large sample size and prevalence of this genus*. Lachnospiraceae Ruminococcus*: β = 0.0128 (0.0122–0.0134), p = 6.8×10^−294^. Higher LE8 adherence was associated with more *Ruminococcus* (a genus of fiber-fermenting bacteria in the Lachnospiraceae family). *Ruminococcaceae Clostridium* (unclassified *Clostridium* in the *Ruminococcaceae* family): β = 0.0583 (0.0401–0.0766), p = 2.9×10^−9^. *Clostridiaceae Clostridium*: β = 0.0283 (0.0092–0.0475), p = 0.013. It should be noted that different *Clostridium* groups appear in different families; some Clostridia can be beneficial, e.g., producers of butyrate*. Ruminococcaceae Ruminococcus*: β = 0.0091 (0.0018–0.0163), p = 0.041. *Victivallaceae Victivallis*: β = 0.0430 (0.0153–0.0707), p = 0.0095. These positive associations suggest that people with healthier lifestyles (higher LE8) have more of these potentially beneficial or fiber-degrading bacteria in their gut.

Conversely, higher LE8 scores were associated with lower abundances of several taxa that could be considered less beneficial or linked to poorer diet: *Lachnospiraceae Clostridium* (a specific genus in Lachnospiraceae, distinct from the *Ruminococcaceae Clostridium* above): β = –0.0521 (95% CI –0.0673 to –0.0368), p = 1.9×10^−10^. This genus was less abundant in high LE8 adherers. *Ruminococcaceae Subdoligranulum*: β = –0.0248 (–0.0378 to –0.0119), p = 9.9×10^−4^. *Coriobacteriaceae Collinsella*: β = –0.0216 (–0.0334 to –0.0009), p = 1.8×10^−3^. *Collinsella* (family Coriobacteriaceae) is a genus sometimes associated with high-fat diets and adverse metabolic profiles; it was found in lower abundance with better LE8 adherence. *Erysipelotrichaceae Clostridium*: β = –0.0261 (–0.0424 to –0.0097), p = 7.7×10^−3^. *Streptococcaceae Streptococcus*: β = –0.0301 (–0.0499 to –0.0102), p = 0.011. Some *Streptococcus* species can be pathobionts in the gut; their lower levels in high LE8 adherers might reflect healthier diet (lower simple sugars, for instance). *Coriobacteriaceae Eggerthella*: β = –0.0345 (–0.0601 to –0.0089), p = 0.026. *Erysipelotrichaceae Catenibacterium*: β = –0.0129 (–0.0136 to –0.0124), p = 7.2×10^−282^. (This extremely significant result likely indicates nearly ubiquitous presence but lower proportion in high LE8; *Catenibacterium* has been associated with high-fat animal-based diets in some studies (Kilburn et al., 2020). These negative associations highlight that certain taxa were less abundant among those with better cardiovascular health.

In summary, the LE8–microbiome association analysis suggests a pattern where higher LE8 adherence (healthier lifestyle) corresponds to a gut microbiome enriched in families like *Barnesiellaceae*, *Ruminococcaceae*, *Lachnospiraceae*, and *Victivallaceae*, and depleted in some *Erysipelotrichaceae*, *Coriobacteriaceae*, *Streptococcaceae*, and specific *Clostridium* groups. Many of the taxa increased with high LE8 are known butyrate producers or associated with fiber-rich diets, whereas those decreased (like *Collinsella*, *Eggerthella*, *Streptococcus*) have been linked to less healthy dietary patterns or inflammation.

It is worth noting that these genera associated with continuous LE8 scores often belong to the same higher-level taxa that differentiate the LE8 groups. For instance, the *Methanobacteriaceae*, *Victivallaceae*, *Barnesiellaceae*, and *Clostridiaceae* families showed higher abundance in high LE8 adherers (as families), consistent with their member genera listed above. On the other hand, families like *Streptococcaceae* and *Coriobacteriaceae* were more abundant in the ModLow LE8 (and their genera *Streptococcus*, *Collinsella* mirrored this in the continuous analysis). We also observed that LE8 score was positively associated with the relative abundance of several major phyla: specifically, *Firmicutes*, *Actinobacteria*, *Proteobacteria*, *and Lentisphaerae* (the latter includes the family *Victivallaceae*). This indicates that broad compositional shifts (not just individual genera) underlie the differences in microbiome with respect to cardiovascular health.

### 3.4 The gut microbiome associates with components of LE8

To disentangle which aspects of the LE8 construct might be driving the microbiome associations, we performed secondary analyses examining each LE8 component in relation to the gut microbiome (**Figure 3**). Using similar MaAsLin2 models, we tested associations between microbial taxa abundance and each component score (diet, physical activity, smoking status, sleep health, BMI, blood glucose, total cholesterol, blood pressure), adjusting for age and sex in each case. **Figure 3** (and **Supplementary Figure S5**) presents a summary heatmap of significant associations between LE8 component scores and microbial taxa (with effect directions). The results suggested that not all components were equally influential on the gut microbiome; a subset of components stood out: Diet score: A healthier diet (higher Dietary score, based on DASH-like diet adherence) was linked to increased abundance of fiber-fermenting bacteria (*Ruminococcaceae*, *Lachnospiraceae genera*) and decreased abundance of some pro-inflammatory taxa (*Streptococcaceae*, *Streptococcus*). This is unsurprising since diet is known to be a major determinant of gut microbiota. Physical activity: Showed trends of association with diversity, but relatively fewer individual taxa passed significance after multiple testing correction. Still, higher physical activity tended to align with microbes associated with leanness or metabolic health (e.g., *Ruminococcus* had a positive trend). Nicotine exposure (smoking): This was one of the top components influencing microbiota. Non-smokers or those with higher nicotine health scores (meaning no tobacco exposure) had higher levels of beneficial genera from *Clostridium* clusters (e.g., *Ruminococcaceae Clostridium, Clostridiaceae Clostridium*) and lower levels of pro-inflammatory *Clostridium* clusters (e.g., *Lachnospiraceae Clostridium*) and some *Eggerthella*. Smoking is known to alter gut microbiota, so this is consistent with prior knowledge. Sleep health: Better sleep (longer duration and quality, as per LE8 definition) was associated with higher abundance of certain SCFA producers. BMI (Body mass index): Although BMI is included in LE8, we considered it separately. Higher BMI (poorer score) correlated with microbiome signatures of obesity (e.g., reduced *Firmicutes*, *Bacteroidetes*, Actinobacteria, more *Ruminococcaceae Clostridium*, etc.), while a healthy BMI (ideal range) showed the opposite. These mirrored known obesity–microbiome patterns. Blood glucose, Blood pressure, Blood lipids: These clinical metrics had somewhat weaker individual associations with microbiome composition, though a few links emerged (e.g., higher blood glucose was related to more *Collinsella*, which aligns with literature on type 2 diabetes microbiome; higher blood pressure had associations with decreased *Ruminococcus and Christesenella*).

**Figure 4:**
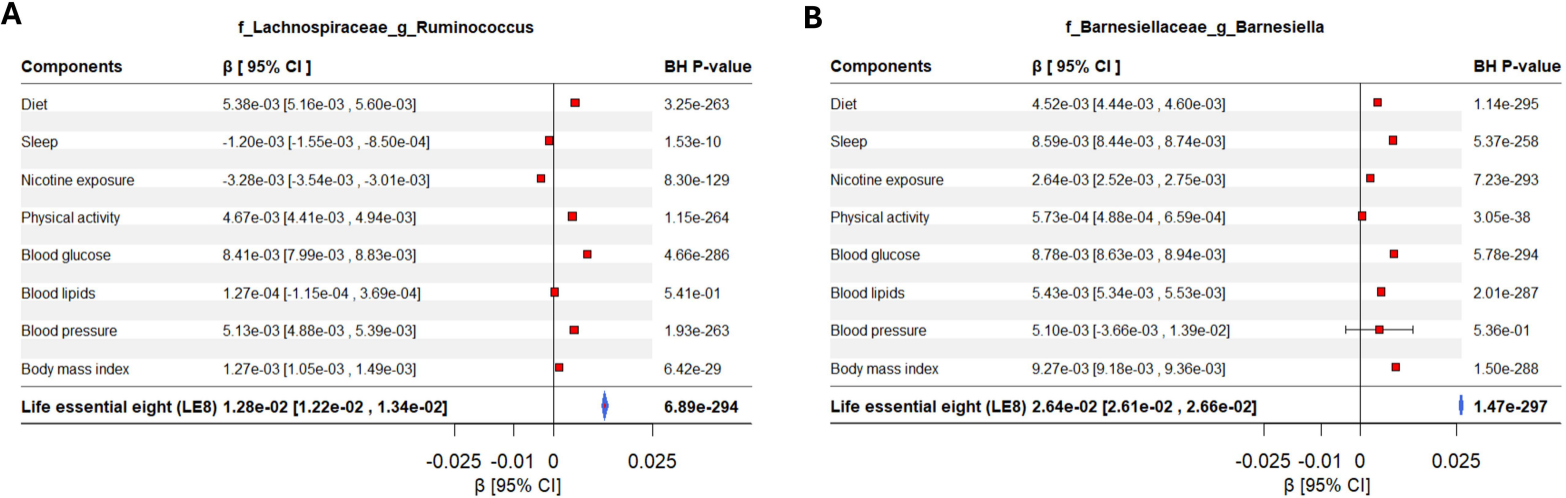
Forest plot displaying the effects of LE8 components in the association between gut microbiome and LE8 adherence. The significant contribution of each metric in the association between gut bacteria and LE8 adherence is clearly observed. For instance, **(A)** *Lachnospiraceae Ruminococcus* and **(B)** *Barnesiellaceae Barnesiella* are mostly influenced by LE8 components but might not respond to blood lipids and blood pressure.

In summary, the top four LE8 components that showed the most prominent relationships with the gut microbiome were nicotine exposure, BMI, diet, and sleep health (**Figure 3** and **Supplementary Figures S6**, **S7**). These four factors likely drive the bulk of the LE8–microbiome association. For example, the genus *Barnesiella* was positively associated with most of these healthy behaviors: it was higher with a healthy diet, in non-smokers, with ideal BMI, and good sleep as showed in **Figure 4A**. Suggesting that *Barnesiella* enrichment might be a common endpoint of a healthy lifestyle (except it showed no clear link with blood pressure). Similarly, a reduced abundance of *Lachnospiraceae Ruminococcus* was tied to worse scores in multiple components (especially sleep and smoking), while its higher abundance aligned with healthier behaviors (**Figure 4B**).

It appears that some bacteria respond to a general “healthy lifestyle” milieu (hence showing up in the composite LE8 analysis), whereas others are more domain-specific. For instance, *Barnesiellaceae Barnesiella* and *Ruminococcaceae Clostridium* are mostly influenced by LE8 components but might not respond to physical activity and blood pressure. Our component analysis provides a nuanced view: health behaviors (including diet, smoking, sleep) and obesity largely affect the abundance of common gut commensals linked to metabolic health, smoking influences a different set of taxa, perhaps via inflammation, and sleep could affect gut rhythms and certain microbe abundances.

These findings underscore that while the composite LE8 score captures an aggregate effect, the underlying drivers in relation to the microbiome are heterogeneous. Nevertheless, the overlap of taxa influenced by multiple healthy behaviors explains why we saw a robust overall LE8 effect.

### 3.5 LE8 adherence is associated with improved cognitive performance

We evaluated the direct relationship between LE8 adherence and cognitive performance in our cohort. Prior studies have suggested a link between better cardiovascular health metrics and better cognition, and our data allowed us to test this within a multivariable framework (Zhou et al., 2023; Clocchiatti-Tuozzo et al., 2024; Liang and Zhang, 2024).

**Figure 5:**
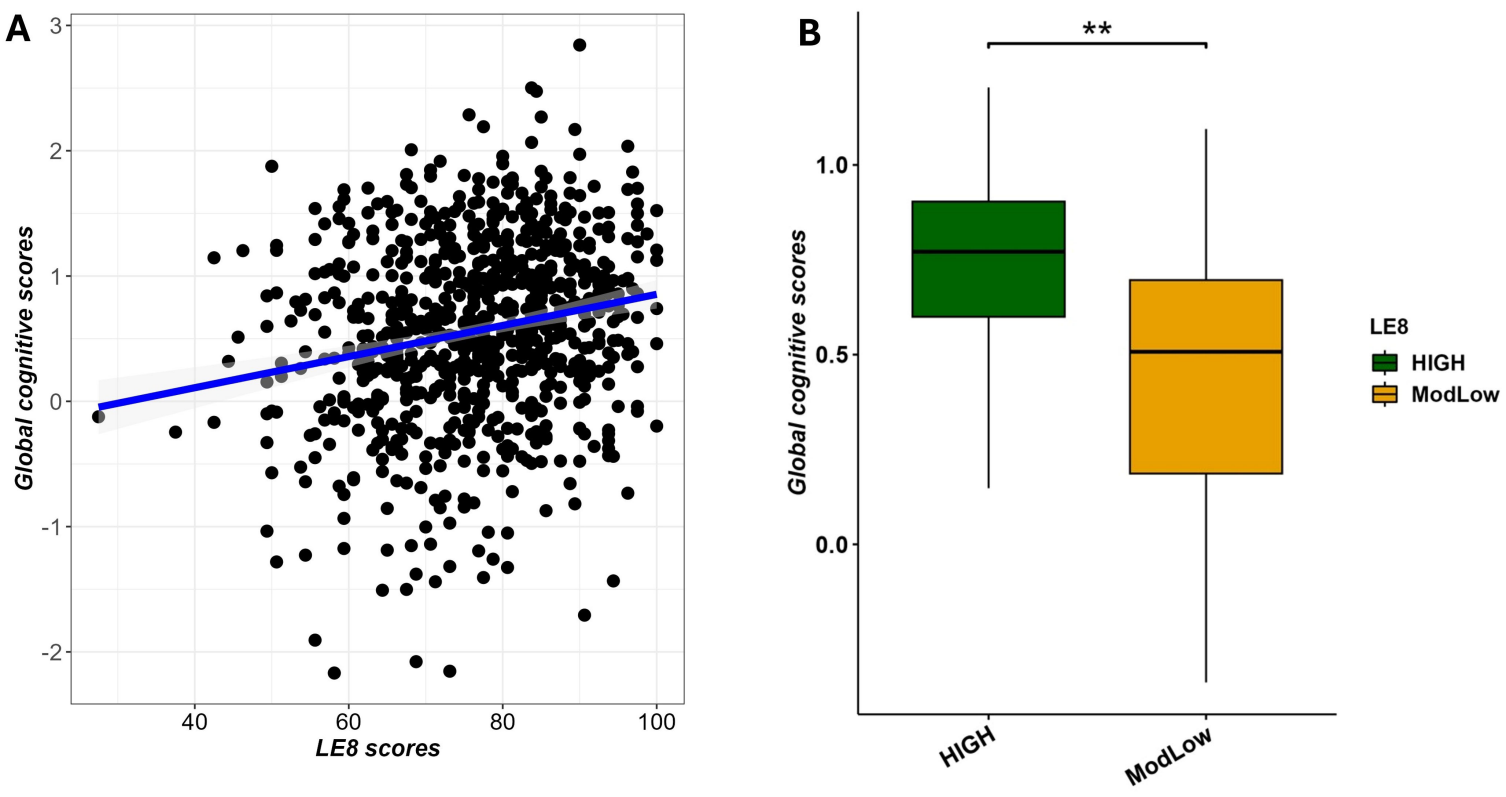
Multivariable association between LE8 adherence and global cognitive scores adjusting for age and sex. **(A)** scatter plot showing a positive correlation between LE8 adherence and GCS. **(B)** Boxplot displaying significant differences in global cognitive scores among LE8 groups. ** denotes 0.001 < adjusted p-value < 0.01.

We conducted multivariable linear regression analysis to examine the relationship between LE8 scores and GCS, adjusting for age, sex, and education. The analysis revealed that higher LE8 scores were significantly associated with increased GCS (β = 0.0047, (95% CI 0.0005–0.0089); adjusted p-value = 2.9×10^−2^), as depicted in **Figure 5A**. In a simpler sense, participants in better cardiovascular health tended to perform better on cognitive testing. **Figure 5A** illustrates this association, showing the regression line of GCS on LE8 score with confidence bands.

To complement the previous analysis, we also performed the same multivariable linear regression with as predictor the binary LE8 status (High vs. ModLow adherence) and the global cognitive scores as outcome. We noted that findings discriminated score of cognitive performance (GCS) from individuals with high adherence compared to those with moderate/low adherence (β = 0.0971 (0.0013–0.1956; p = 7.4×10^−3^), as displayed in **Figure 5B**.

Collectively, these results align with the hypothesis that maintaining ideal cardiovascular health (as summarized by LE8) is associated with better cognitive function. They set the stage for investigating whether the gut microbiome plays a role in this link.

### 3.6 Association between the gut microbiome and cognitive performance

We next turned to the connection between gut microbiome features and cognitive outcomes (independent of LE8). First, we evaluated microbiome diversity in relation to cognitive performance. After confirming GCS was approximately normal and dichotomizing as described, we found that participants with poor cognition (lowest quintile GCS) had significantly lower alpha-diversity compared to those with normal cognition. In **Supplementary Figure S8A**, the Chao1 and Shannon indices are both lower in the Poor cognition group; for example, median Shannon was 3.5 in Poor vs. 3.7 in Normal (Wilcoxon p = 0.0066). Chao1 richness median was 149.2 vs. 160.1 (p = 0.0012). This suggests that lower cognitive performance was associated with a less diverse gut microbiome. We also performed adjusted linear models for diversity and GCS (with age, sex, BMI, education, time difference as covariates) and found a modest but significant positive association: e.g., each 1-unit higher GCS was associated with +0.07 higher Shannon index (p = 0.025). These results mirror the LE8-diversity findings, now in the context of cognition.

Beta-diversity analysis similarly indicated that the overall microbiome composition differed between the Normal vs. Poor cognition groups. PERMANOVA on Bray–Curtis distances gave p = 1×10^−4^ (unadjusted) for cognitive group differences, and p = 1×10^−3^ after adjusting for age, sex, BMI, education, and time interval. PCoA plots (**Supplementary Figure S8B**) show some separation of the two cognition groups, albeit with overlap (which is expected given that many factors influence microbiome beyond cognition). These findings imply a global association where cognitively poorer individuals, as a group, have a shifted gut microbiome structure relative to cognitively normal individuals. **Supplementary Figures S9** and **S10** provide stacked barplots of the microbiome composition in normal vs. poor cognition groups at broad taxonomic levels, illustrating these differences.

**Figure 6:**
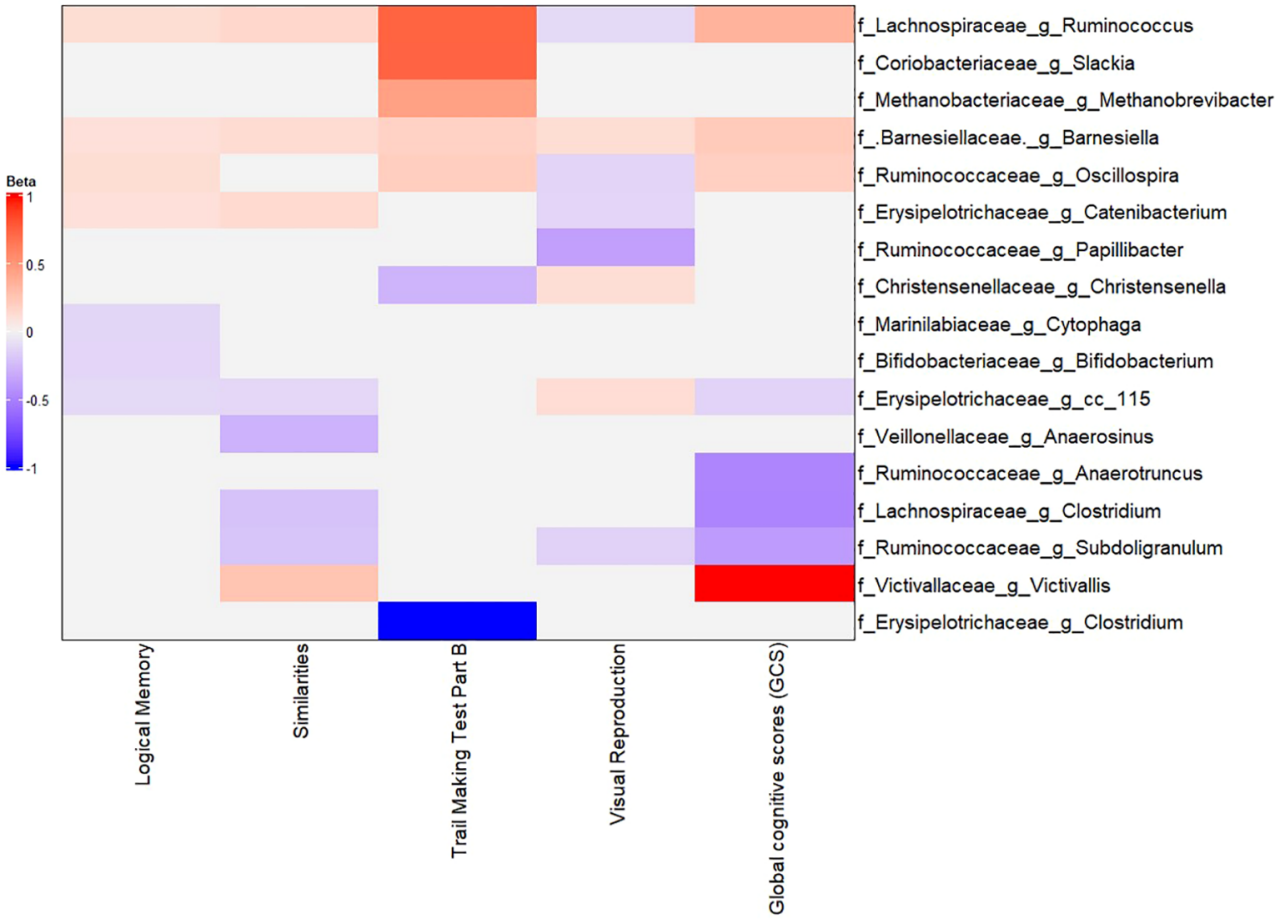
Multivariable association between gut microbiota and global cognitive scores. Heatmap depicting the significant (adjusted p-value< 0.05) genera associated with GCS along with the scores of tests composing GCS.

We then identified specific taxa associated with cognitive performance by using MaAsLin2 models as described in the Methods section. **Figure 6** (with **Supplementary Figures S11–S12**) summarizes these associations. Several interesting patterns emerged: Higher (better) cognitive performance (GCS) was positively associated with the abundance of certain genera, many overlapping with those identified in the LE8 analysis: *Lachnospiraceae Ruminococcus*: Higher GCS correlated with higher abundance of this genus (β = 0.2523 (95%CI 0.2423–0.2623), p = 5.3×10^−281^). This genus was also positively linked to LE8; here we see it also aligns with better cognition. *Barnesiellaceae Barnesiella*: β = 0.1219 (0.1182–0.1255), p = 4.4×10^−267^. Again, *Barnesiella* appears as a beneficial taxon correlated with both healthy lifestyle and better cognition. *Ruminococcaceae Oscillospira*: β = 0.0943 (0.0663–0.1223), p = 3.0×10^−10^. *Oscillospira* (often associated with leanness and high-fiber diet) was more abundant in those with higher GCS. *Victivallaceae Victivallis*: β = 0.6558 (0.1840–1.1277), p = 0.029. This genus was at higher level in the good cognition group as well.

In addition, at the family level, *Methanobacteriaceae*, *Christensenellaceae*, *Rikenellaceae*, *Ruminococcaceae*, and *Victivallaceae* all had positive associations with GCS (these families are typically considered part of a healthy gut microbiome profile in literature, often linked to low inflammation or positive metabolic traits). At the phylum level, better cognition was associated with higher relative abundances of *Lentisphaerae* and *Firmicutes*, and *OD1* (*Parcubacteria*).

On the other hand, lower cognitive performance (poor GCS) was associated with higher abundances of some taxa: *Erysipelotrichaceae cc_115*: β = –0.0396 (−0.0579 to −0.0213), p = 1.3×10^−4^. This indicates higher *cc_115* (an uncultured genus of *Erysipelotrichaceae*) in those with lower GCS. *Lachnospiraceae Clostridium*: β = –0.3984 (−0.6608 to −0.1361), p = 0.014. This same genus was higher in the ModLow LE8 score and appears again with poorer cognition. *Ruminococcaceae Subdoligranulum*: β = –0.2950 (−0.5155 to −0.0775), p = 0.037. Higher *Subdoligranulum* in poor cognition; interestingly this genus also decreased with higher LE8. *Ruminococcaceae Anaerotruncus*: β = –0.3881 (−0.6889 to −0.0871), p = 0.047. *Anaerotruncus* higher in poor cognition. Additionally, phyla *OD1*, *Lentisphaerae*, and *Firmicutes* had positive associations with GCS (meaning they were lower in poor cognition).

To summarize, the gut microbiome of individuals with better cognitive performance was enriched in SCFA-producing, fiber-degrading, and putatively anti-inflammatory taxa (like *Ruminicoccus*, *Barnesiella*, *Oscillospira*, *Christensenellaceae* family) and had fewer pro-inflammatory or dysbiosis-associated taxa (like certain *Clostridium* clusters). These patterns mirror those seen with LE8 adherence, which is suggestive of a common pathway: the same beneficial bacteria that are promoted by a healthy lifestyle might also promote or reflect better neurological health.

### 3.7 The gut microbiome associates with neuropsychological tests comprising the GCS

To drill down further, we explored whether certain bacteria were particularly associated with specific cognitive domains or tests (rather than the composite GCS alone). We conducted exploratory analyses relating taxa abundance to each of the four main cognitive test scores (Logical Memory, Visual Reproduction, Similarities, Trail Making B). These were adjusted for the same covariates. While a full description is beyond scope, a few noteworthy observations include: (a) The reduced abundance of *Barnesiella* (which was strongly linked with poor GCS) was specifically associated with lower scores on Similarities, Visual Reproduction, Logical Memory, and slower Trail Making B (i.e., all components of GCS). In other words, individuals with reduced abundance of *Barnesiella* tended to perform worse across multiple cognitive domains. (b) Lower abundance of *Lachnospiraceae Ruminococcus* was significantly associated with lower scores on Logical Memory and Similarities, and higher (worse) time on Trail making part B, though interestingly it was associated with higher Visual Reproduction in this analysis (perhaps reflecting a complex relationship or multiple strains). (c) *Lachnospiraceae Clostridium* and *Ruminococcaceae Subdoligranulum*, which were higher in poor performers, each showed negative associations with some cognitive tests (e.g., more *Subdoligranulum* linked to worse memory and executive function). (d) Some taxa had differential associations: for example, *Bifidobacterium* (*Actinobacteria* phylum) had a positive correlation with memory scores but wasn’t a top mediator in overall GCS.

These results (depicted partially in **Figure 6**) suggest that the microbiome-cognition link is not driven by a single domain but rather a general effect across cognitive functions. *Barnesiella*, in particular, stands out as consistently associated with better performance on all tests, reinforcing its potential role in overall cognitive health.

### 3.8 Mediation analysis: gut microbiome in the LE8–cognition link

**Figure 7:**
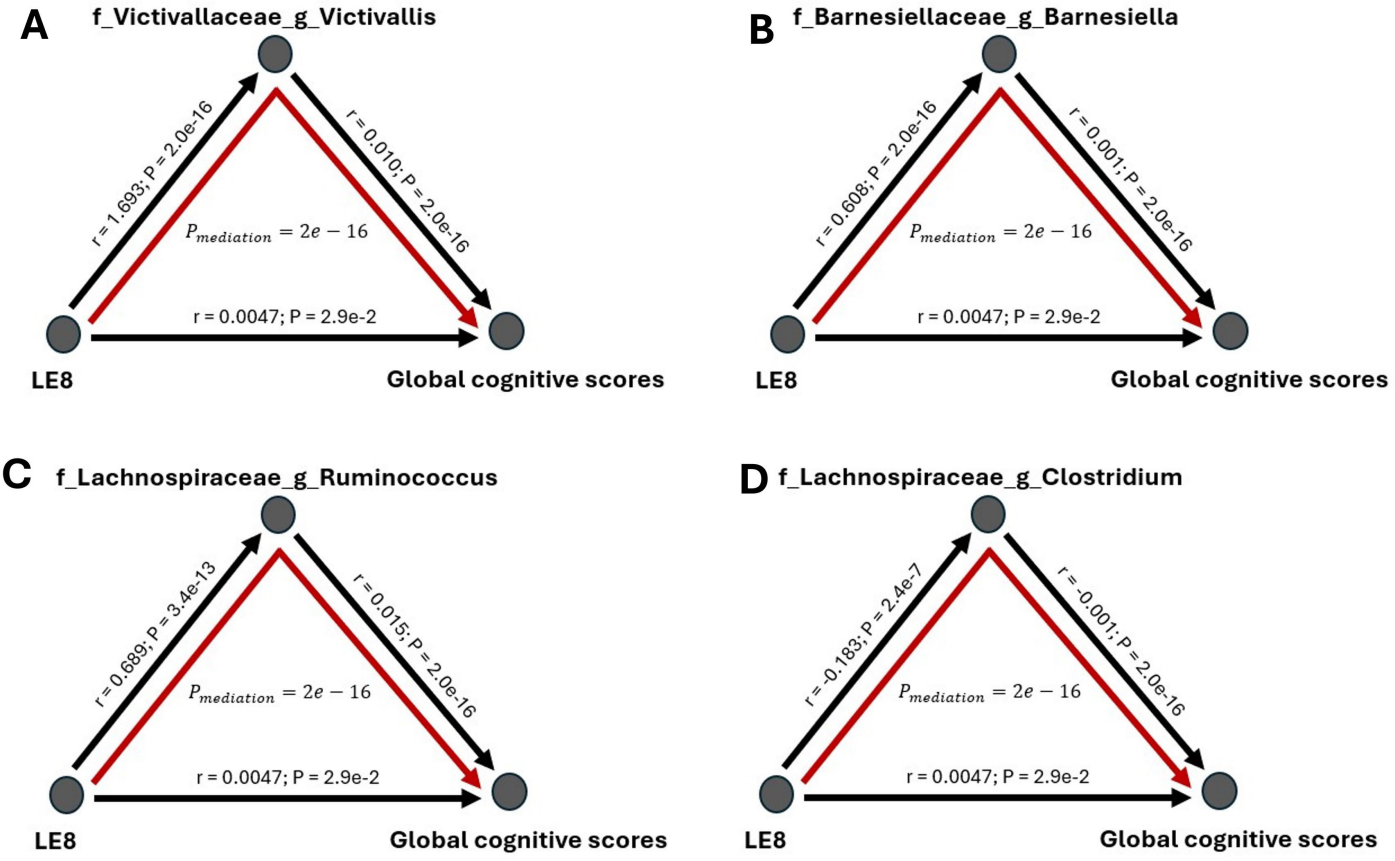
Mediation analysis of gut microbiome on the association between LE8 adherence scores and global cognitive scores. The mediation effect of bacteria **(A)** *Victivallaceae Victivallis*, **(B)** *Barnesiellaceae Barnesiella*, **(C)** *Lachnospiraceae Ruminococcus*, and **(D)** *Lachnospiraceae Clostridium* on the association of lower cognition with moderate and low LE8 adherence.

**Figure 8:**
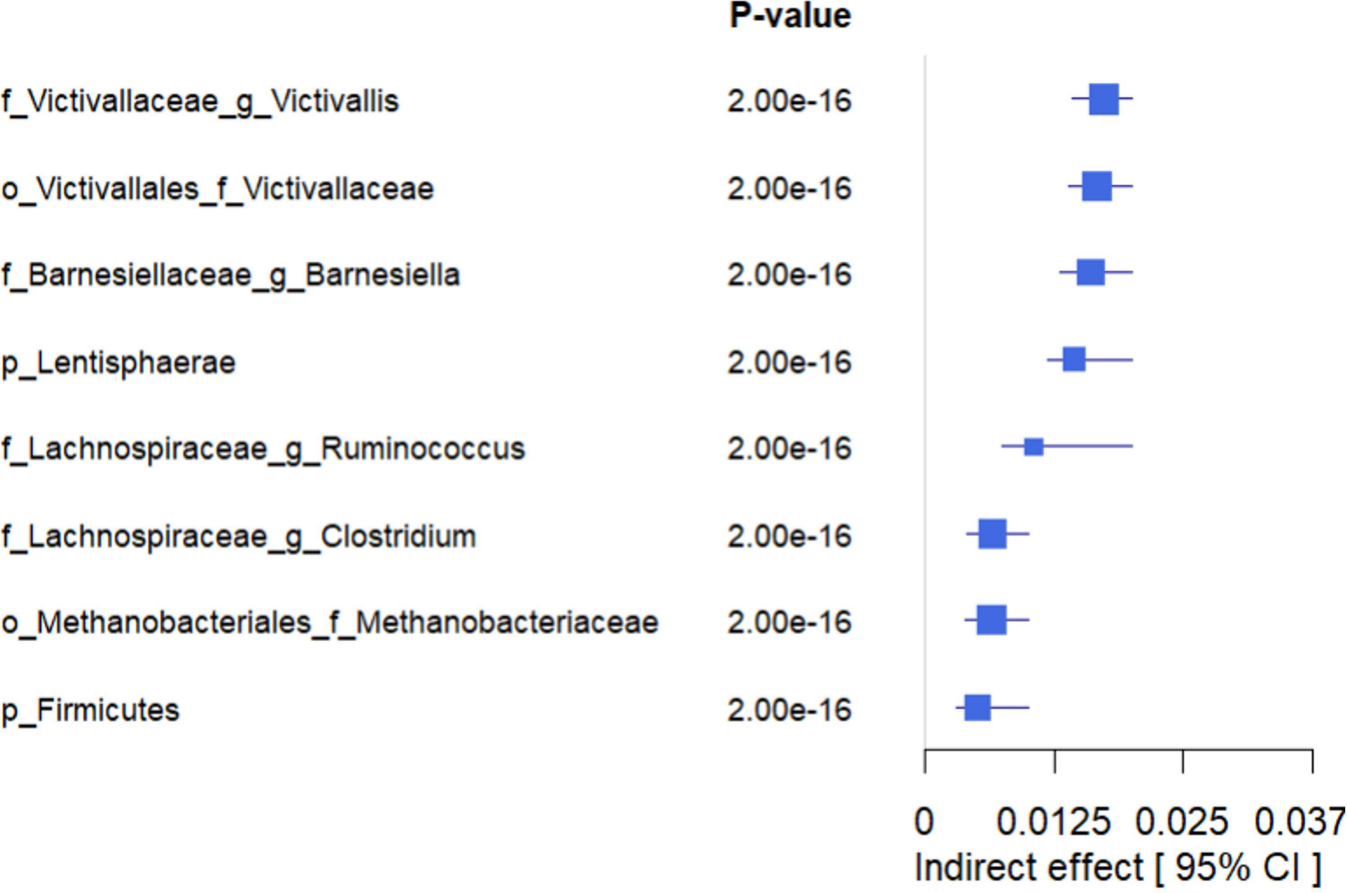
Forest plot showing the estimated indirect effects of bacteria that mediate the association between higher adherence to LE8 with better cognition. As we can see, the top 4 strongest IDE include *Victivallaceae Victivallis, Victivallaceae, Barnesiellaceae Barnesiella, and Lentisphaerae*.

Finally, we formally tested whether the gut microbiome mediates the relationship between LE8 adherence and cognitive performance. From the above analyses, we identified overlapping taxa that were associated with both LE8 and GCS. These overlapping taxa are prime candidates for mediation. Notably, *Victivallaceae Victivallis*, *Barnesiellaceae Barnesiella*, *Lachnospiraceae Ruminococcus*, *Lachnospiraceae Clostridium*, *Victivallaceae (unclassified genus)*, *Methanobacteriaceae (family level)*, *Lentisphaerae (phylum level)*, and *Firmicutes (phylum level)* were in this overlapping set. For each candidate, we conducted a mediation analysis using the approach described above. We report the mediation effect sizes (indirect effects, IDE) and significance in **Figure 7** and **Figure 8** (with details in **Supplementary Figure S13** for all tested mediators).

The mediation analyses revealed that several bacterial taxa significantly mediated the association between LE8 adherence and GCS: *Victivallaceae Victivallis*: This genus had one of the strongest mediation effects. High LE8 adherence was associated with increased *Victivallis*, which in turn was associated with higher GCS. The indirect effect (IDE) was 0.0173 (95% CI 0.0143–0.0200), with p_boot< 0.001, indicating a significant mediation. *Victivallaceae (unclassified family)*: IDE = 0.0166 (95% CI 0.0139–0.0197), p_boot< 0.001. Similar interpretation: part of LE8’s effect on GCS goes through changes in the *Victivallaceae* family. *Barnesiellaceae Barnesiella*: IDE = 0.0160 (95% CI 0.0130–0.0190), p_boot< 0.001. This suggests *Barnesiella* accounts for a notable portion of the LE8–cognition link; i.e., LE8 adherence might improve cognition partly by increasing *Barnesiella* abundance, which has neuroprotective associations. *Lentisphaerae (phylum)*: IDE = 0.0144 (95% CI 0.0118–0.0170), p_boot< 0.001. Many of the *Victivallaceae* belong to *Lentisphaerae*, so this aligns with the above. *Methanobacteriaceae (family)*: IDE also significant (~0.012, p_boot< 0.001); *Methanobacteriaceae* (includes *Methanobrevibacter*) were higher with LE8 and with good cognition. *Firmicutes (phylum):* Showed a smaller but significant mediation, indicating that broad community shifts in *Firmicutes* abundance could explain part of the effect. *Lachnospiraceae Ruminococcus*: This genus mediated a portion as well (IDE ~0.0065, p_boot< 0.001). Conversely, *Lachnospiraceae Clostridium* showed a negative link effect: higher LE8 was associated with lower *Lachnospiraceae Clostridium*, which was associated with higher cognition (since this genus is harmful), meaning that reducing this taxon is one pathway by which LE8 adherence might benefit cognition.

**Figure 8** highlights the top four mediating bacteria by absolute effect size: *Victivallis*, *Victivallaceae*, *Barnesiella*, and *Lentisphaerae*. These each contributed an indirect effect on the order of 0.014–0.017 in GCS units. Given that the total effect of LE8 on GCS (per 10 points of LE8) was ~0.047, an indirect effect of ~0.016 suggests that roughly one-third of the LE8→cognition relationship could be explained by that single mediator. Cumulatively, considering all significant microbial mediators together, they potentially account for a substantial portion of the association (though they are not independent, so we refrain from simple summing).

## 4 Discussion

This study provides a comprehensive examination of the relationships between adherence to Life’s Essential 8 (LE8), the gut microbiome, and cognitive performance, utilizing robust data from the Framingham Heart Study. Our findings emphasize the critical role of cardiovascular health metrics in shaping the gut microbiota and their downstream effects on cognitive resilience.

We found that higher LE8 adherence (better overall cardiovascular health) is associated with a more diverse and even gut microbiome, evidenced by significant differences in alpha-diversity between high and moderate adherence groups. This suggests that engaging in healthy behaviors (diet, exercise, not smoking, adequate sleep, etc.) fosters a rich gut microbial ecosystem. We also observed distinct clustering of microbial profiles by LE8 group in beta-diversity analyses, indicating that LE8 adherence influences overall microbial community structure. Notably, the increase in beneficial taxa such as *Faecalibacterium*, *Barnesiella*, and *Ruminococcus* in the high adherence group suggests that LE8 adherence promotes a gut microbiota composition favorable for health. Many of these bacteria are known to produce short-chain fatty acids and have anti-inflammatory properties, which could have systemic benefits.

Concurrently, our analyses demonstrated that better LE8 adherence correlates with superior cognitive performance. This aligns with prior research linking cardiovascular health to cognition (De Moraes et al., 2024; Xu et al., 2024). In our cohort, individuals with high LE8 scores performed better on a composite cognitive measure (GCS) even after accounting for age, sex, and education. This association underscores how healthy lifestyle factors that benefit the heart and vasculature likely also support brain health, potentially through improved cerebral blood flow, reduced vascular risk, and lower inflammation.

A central novel contribution of our work is the mediation analysis, which highlights the gut microbiome as a key intermediary linking LE8 adherence to cognitive performance. We identified specific bacterial taxa (e.g., *Barnesiella*, *Ruminococcus*, *Victivallis*, among others) that statistically mediate part of the LE8–cognition relationship. These mediating taxa are notable for their functional profiles: for instance, *Barnesiella* and *Ruminococcus* are genera known for anti-inflammatory effects and production of SCFAs such as butyrate, which supports gut barrier integrity and may exert neuroprotective effects via the gut–brain axis (Huang et al., 2024; Zhu et al., 2024b; Guo et al., 2025). Our mediation findings suggest that a portion of the cognitive benefit associated with a healthy lifestyle may be transmitted through an enriched presence of these beneficial microbes. In other words, a healthy lifestyle might lead to a healthier microbiome, which in turn produces metabolites (like SCFAs) and other signals that promote brain health. We also observed that a potentially deleterious genus, *Lachnospiraceae Clostridium*, was reduced with better lifestyle and that this reduction was associated with better cognition; this could indicate that avoiding dysbiotic microbes (often linked to pro-inflammatory states) is another pathway through which healthy habits protect the brain. Taxonomic analysis and prior literature suggest that these beneficial genera may contribute to neuroprotective mechanisms, potentially through the production of short-chain fatty acids (SCFAs), reinforcing the biological plausibility of the mediation results.

The implications of these findings are significant for public health and clinical practice. LE8 metrics represent modifiable lifestyle factors that can be improved through interventions such as dietary changes, exercise programs, smoking cessation, and better sleep hygiene. Our study suggests that improving these behaviors not only benefits cardiovascular outcomes but also may confer cognitive benefits, with the gut microbiome playing a role in that process. This raises the intriguing possibility of microbiome-targeted strategies to amplify or complement lifestyle interventions. For example, if certain gut bacteria (like *Barnesiella* or *Faecalibacterium*) are key mediators, then interventions like probiotics, prebiotic supplements, or diets specifically designed to increase those taxa could potentially enhance cognitive resilience. The identification of specific microbial taxa as mediators also underscores the potential for microbiome-based biomarkers; one could envision using the presence or abundance of these bacteria as indicators of an individual’s trajectory of brain health in response to lifestyle changes.

Our results also highlight the importance of a holistic approach to health. We showed that by adhering to LE8 metrics, individuals not only improve cardiovascular health but also potentially enhance cognitive resilience through microbiome-mediated mechanisms. This reinforces the concept that “what’s good for the heart is good for the brain,” and adds that the gut microbiome is an important piece of that puzzle. From a clinical perspective, it provides further incentive to encourage patients to adopt heart-healthy lifestyles, as the benefits likely extend to brain aging and the delay of cognitive decline.

While our study provides valuable insights, it also raises important questions for future research. One limitation is the cross-sectional design, which precludes definitive conclusions about causality or directionality. Longitudinal studies will be crucial to determine the temporal sequence and causal relationships among LE8 adherence, microbiome changes, and cognitive outcomes. For example, do improvements in lifestyle lead to microbiome shifts that then lead to cognitive improvements (causality as we hypothesize), or could baseline microbiome composition influence one’s ability to maintain a healthy lifestyle or directly impact cognition (reverse or bidirectional effects)? Only through prospective tracking and intervention studies can these questions be answered.

Additionally, advanced multi-omics and sequencing techniques should be employed in future work. While 16S rRNA sequencing allowed us to profile microbiota composition, shotgun metagenomic sequencing could provide functional information about microbial pathways (e.g., genes for SCFA synthesis, neurotransmitter metabolism) that might be mediating cognitive effects (Lai et al., 2021; Liang et al., 2022). Metabolomic profiling of microbial metabolites in blood or cerebrospinal fluid would also greatly enhance understanding of the gut–brain biochemical communication. Our finding of SCFA-producing taxa being beneficial suggests SCFAs as candidates, but many other microbial metabolites (tryptophan metabolites, bile acids, etc.) could also play a role.

Another avenue is to conduct randomized controlled trials targeting the microbiome to see if cognitive outcomes can be improved. For instance, a trial could test whether a high-fiber diet or a probiotic (designed to boost *Faecalibacterium/Barnesiella*) in individuals with low LE8 scores can improve their cognition over time, compared to a control group (Prokopidis et al., 2022; Azuma et al., 2023). If successful, that would strongly support the causality of the microbiome’s role. Moreover, intervention studies could explore whether combining lifestyle modification with microbiome modulation yields additive or synergistic benefits for cognitive health.

In conclusion, our study underscores the intertwined nature of cardiovascular wellness, gut microbial homeostasis, and brain health. By demonstrating that LE8 adherence is linked to a favorable gut microbiome and better cognitive performance, and that these links intersect through specific microbial players, we contribute to a growing recognition of the gut–heart–brain axis. These findings encourage integrated approaches to disease prevention: addressing cardiovascular risk factors, nurturing a healthy gut microbiota (through diet and possibly probiotics/prebiotics), and monitoring cognitive health together. Such an approach could be especially relevant in aging populations at risk for dementia, where lifestyle interventions and microbiome-based therapies might together mitigate cognitive decline.

## 5 Conclusion

In summary, this study highlights the interconnected relationship between cardiovascular health, gut microbiome diversity, and cognitive function. We found that higher adherence to Life’s Essential 8 metrics is associated with favorable gut microbial profiles and enhanced cognitive performance, with the gut microbiome potentially serving as a critical mediator. These findings emphasize the importance of integrated lifestyle interventions that address cardiovascular and cognitive health simultaneously. By adopting heart-healthy behaviors (balanced diet, regular exercise, no smoking, adequate sleep, etc.), individuals may cultivate a gut microbiome environment that supports brain health, thereby potentially reducing the risk of cognitive decline. To validate and extend these results, future research should prioritize longitudinal studies and randomized controlled trials to explore the causal pathways and to test microbiome-targeted interventions as a means of preserving cognitive function. Our work underscores a proactive approach to optimizing both physical and mental health outcomes by targeting modifiable factors at multiple levels—lifestyle behaviors, gut microbial ecology, and vascular health—ultimately contributing to healthier aging and reduced burden of dementia.

## Data Availability

Publicly available datasets were analyzed in this study. This data can be found here: All data supporting the findings of this study are publicly accessible through dbGap (Study ID: phs000007.v32.p13, https://www.ncbi.nlm.nih.gov/projects/gap/cgi-bin/study.cgi?study_id=phs000007.v32.p13).
