# Supplementary material for: Adherence to Life’s Essential 8 enhances gut microbiota diversity and cognitive performance
Source: Front Microbiomes. 2025 Jun 23;4:1592023. doi: 10.3389/frmbi.2025.1592023 (PMC12993607; doi:10.3389/frmbi.2025.1592023)
Supplement: Supplementary file 1 [file SupplementaryFile1.docx]

Adherence to Life's Essential 8 Enhances Gut Microbiota Diversity and Cognitive Performance

Yannick N. Wadop^1^, Jazmyn Muhammad^1^, Rebecca Bernal^1^, Claudia L. Satizabal^1,2,3,4^, Alexa Beiser^3,4,5^, Ramachandran S. Vasan^3,6,7,8,9,10,11^, Ramnik Xavier^12,13,14^, Tiffany Kautz^1,15^, Sudha Seshadri^1,3,4,16^, Jayandra Jung Himali^1,2,3,4,5^, Bernard Fongang^1,2,17,*^

^1^Glenn Biggs Institute for Alzheimer’s and Neurodegenerative Diseases, University of Texas Health Science Center at San Antonio, San Antonio, TX USA

^2^Department of Population Health Sciences, University of Texas Health Science Center at San Antonio, San Antonio, TX USA

^3^Framingham Heart Study, Framingham, MA USA

^4^Department of Neurology, Boston University Chobanian & Avedisian School of Medicine, Boston, MA USA

^5^Department of Biostatistics, Boston University School of Public Health, Boston, MA USA

^6^Department of Medicine, Section of Cardiovascular Medicine, Boston Medical Center, Boston University School of Medicine, Boston, MA USA

^7^Department of Medicine, Section of Preventive Medicine and Epidemiology, Boston University School of Medicine, Boston, MA USA

^8^Department of Epidemiology, Boston University School of Public Health, Boston, MA USA

^9^Boston University’s Center for Computing and Data Sciences, Boston, MA USA

^10^The University of Texas School of Public Health in San Antonio, San Antonio, TX USA

^11^The Long School of Medicine, University of Texas Health Science Center, San Antonio, TX USA

^12^Broad Institute of MIT and Harvard, Cambridge, MA, USA.

^13^Center for Microbiome Informatics and Therapeutics, Massachusetts Institute of Technology, Cambridge, MA, USA.

^14^Center for Computational and Integrative Biology and Department of Molecular Biology, Massachusetts General Hospital and Harvard Medical School, Boston, MA, USA.

^15^Department of Medicine, University of Texas Health Science Center at San Antonio, San Antonio, TX USA

^16^Department of Neurology, University of Texas Health Science Center at San Antonio, San Antonio, TX USA

^17^Department of Biochemistry and Structural Biology, University of Texas Health Science Center at San Antonio, San Antonio, TX USA

* Correspondence:

Corresponding Author

fongang@uthscsa.edu

Supplementary Material

1. **Relationship between LE8 score and each individual component of LE8**


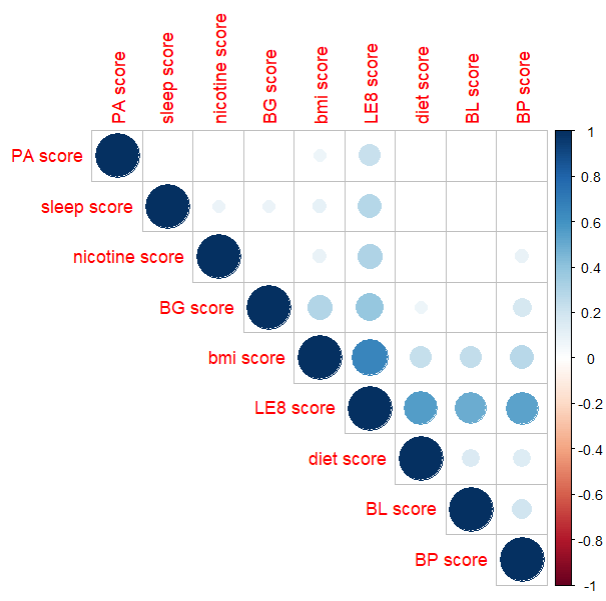


**Figure S1.** Spearman correlation exhibiting the relationship between LE8 score and each individual component of LE8.

1. **Taxonomic profiling from stool samples of FHS subjects in LE8 adherence groups**


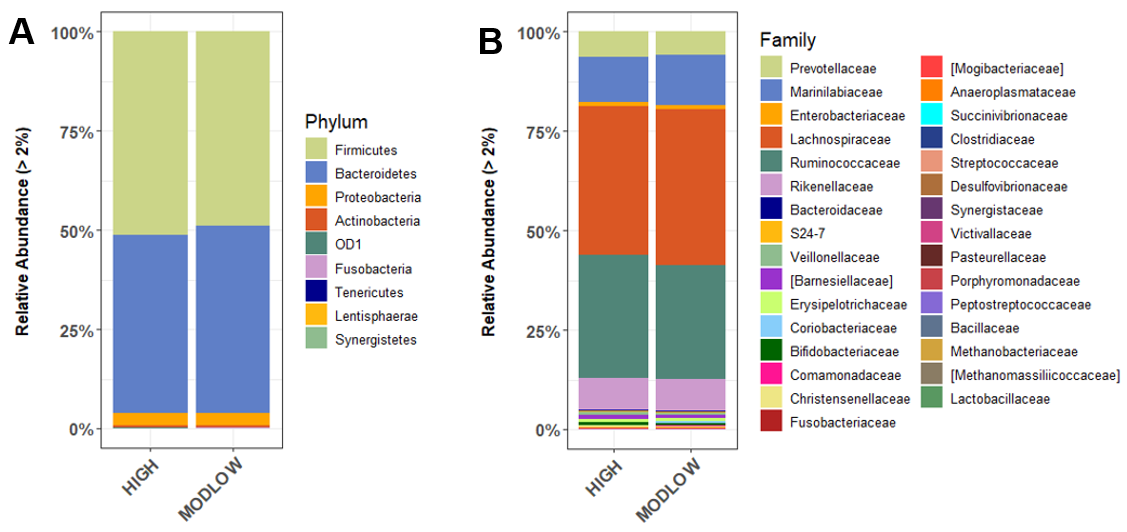


**Figure S2.** Stacked plot showing the microbiome profiles of predominant taxa in the stool samples of high and ModLow LE8 adherence groups at the (A) phylum and (B) family levels.


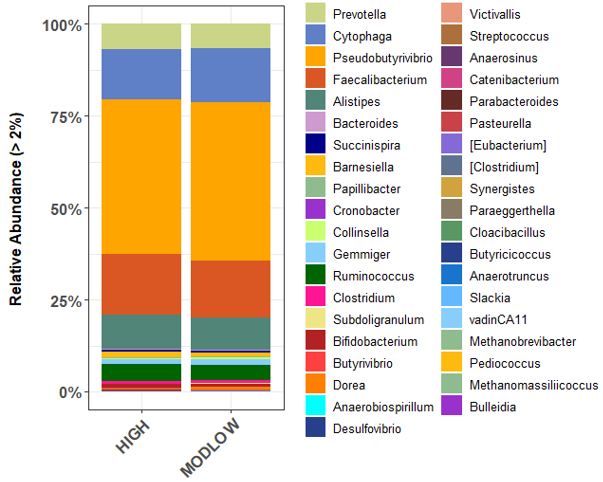


**Figure S3.** Stacked plot showing the microbiome profiles of predominant taxa in the stool samples of high and ModLow LE8 adherence groups at the genus level.

**
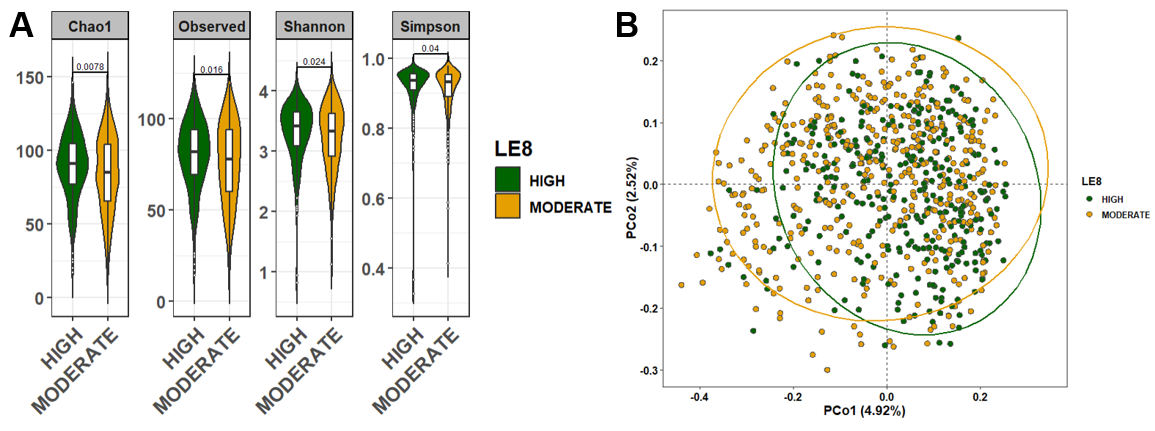
**

**Figure S4.** Comparison of the microbial community diversity of samples between high and moderate LE8 adherence groups. (A) The α-diversity analysis through calculation of Chao1, Observed, Shannon, and Simpson indexes. The tests of difference in microbial diversity at the OTU level between both LE8 groups were based on Wilcoxon test. (B) Beta diversity on samples represented by PCoA using Bray-Curtis dissimilarity. The p-value of PERMANOVA test is 1.0e-4.

1. **LE8 Adherence and Gut Microbiome**


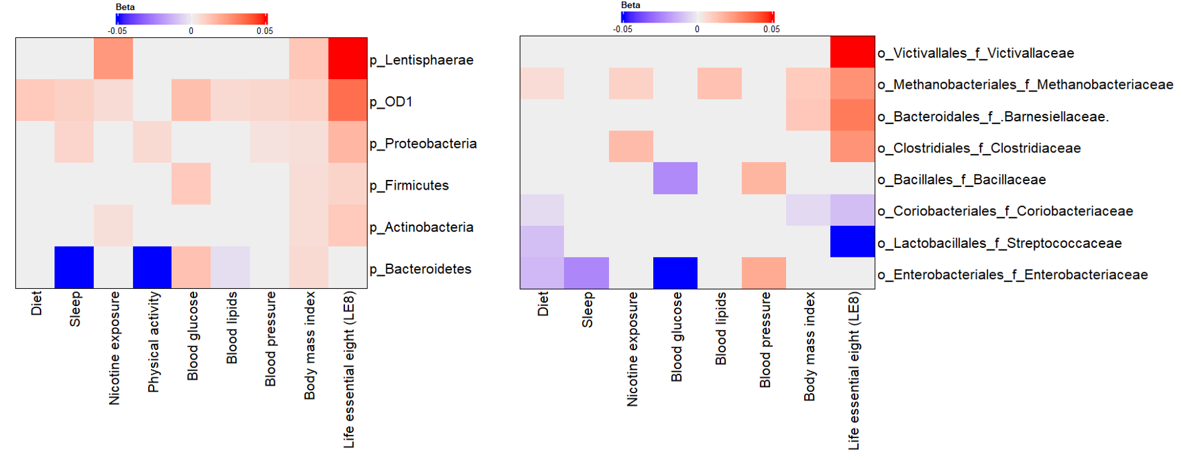


**Figure S5.** Multivariable association between gut microbiota and LE8 adherence. Heatmap depicting the significant (adjusted p-value < 0.05) taxa associated with LE8 adherence along with its components at the phylum (left) and family (right).

1. **Forest plots exhibiting the effects of LE8 components on LE8-gut microbiome relationships**

**
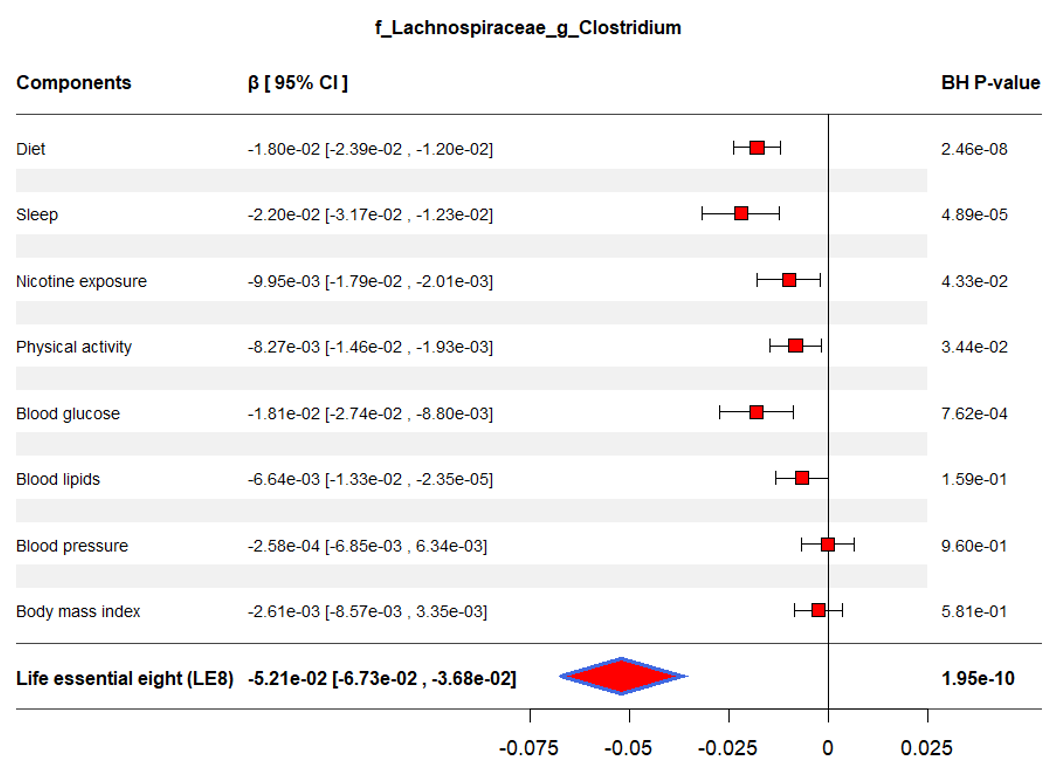
**

**Figure S6.** Forest plot displaying the effects of LE8 components in the association between LE8 adherence and abundance of *Lachnospiraceae Clostridium*

**
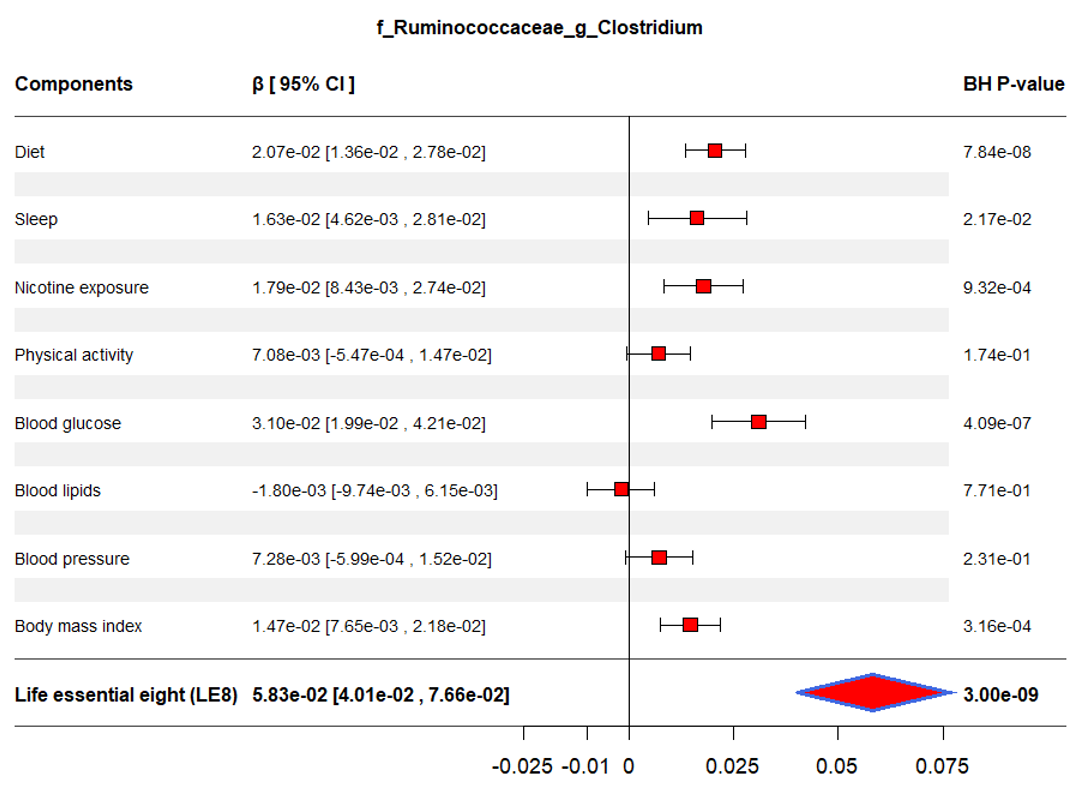
**

**Figure S7.** Forest plot displaying the effects of LE8 components in the association between LE8 adherence and abundance of *Ruminococcaceae Clostridium*.

1. **Diversity analysis showed differences across GCS groups**


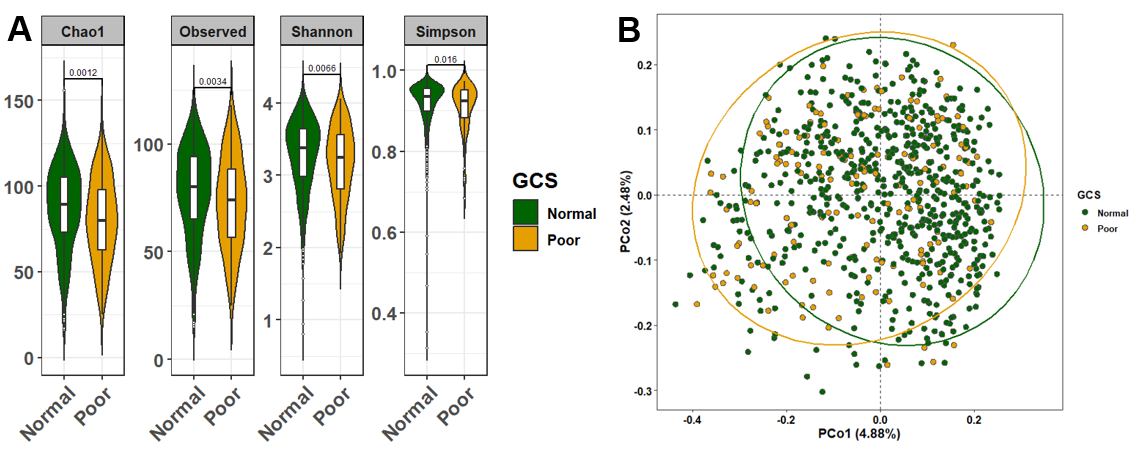


**Figure S8.** Comparison of the microbial community diversity of samples between the normal and poor groups of GCS. (A) The α-diversity analysis through calculation of Chao1, Observed, Shannon, and Simpson indexes. The tests of difference in microbial diversity at the OTU level between both GCS groups were based on the Wilcoxon test. (B) Beta diversity on samples represented by PCoA using Bray-Curtis dissimilarity. The p-value of PERMANOVA test is 1.0e-4.

1. **Taxonomic profiling from stool samples of FHS subjects in GCS groups**


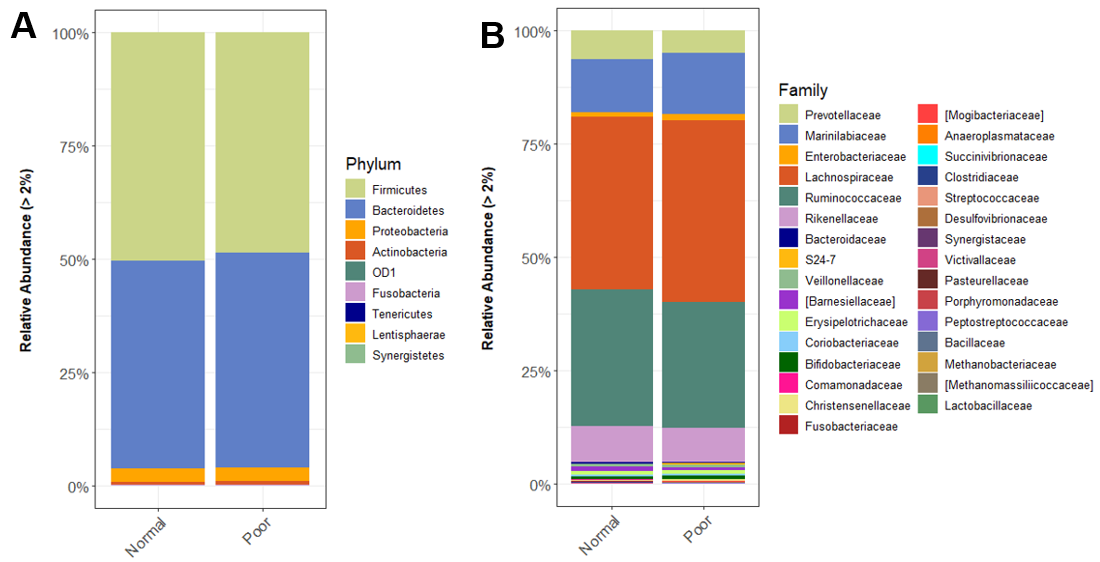


**Figure S9.** Stacked plot showing the microbiome profiles of predominant taxa in the stool samples of normal and poor GCS groups at the phylum and family levels.


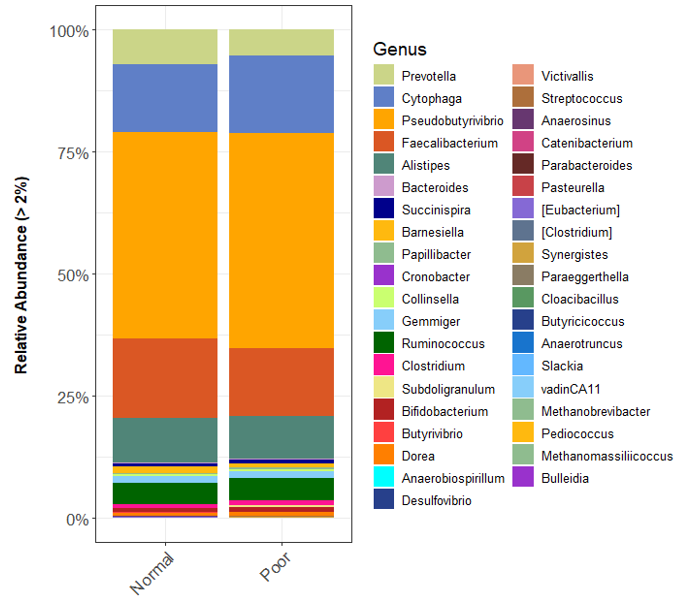


**Figure S10.** Stacked plot showing the microbiome profiles of predominant taxa in the stool samples of normal and poor GCS groups at the genus level.

1. **Gut Microbiome and Cognitive Performance**


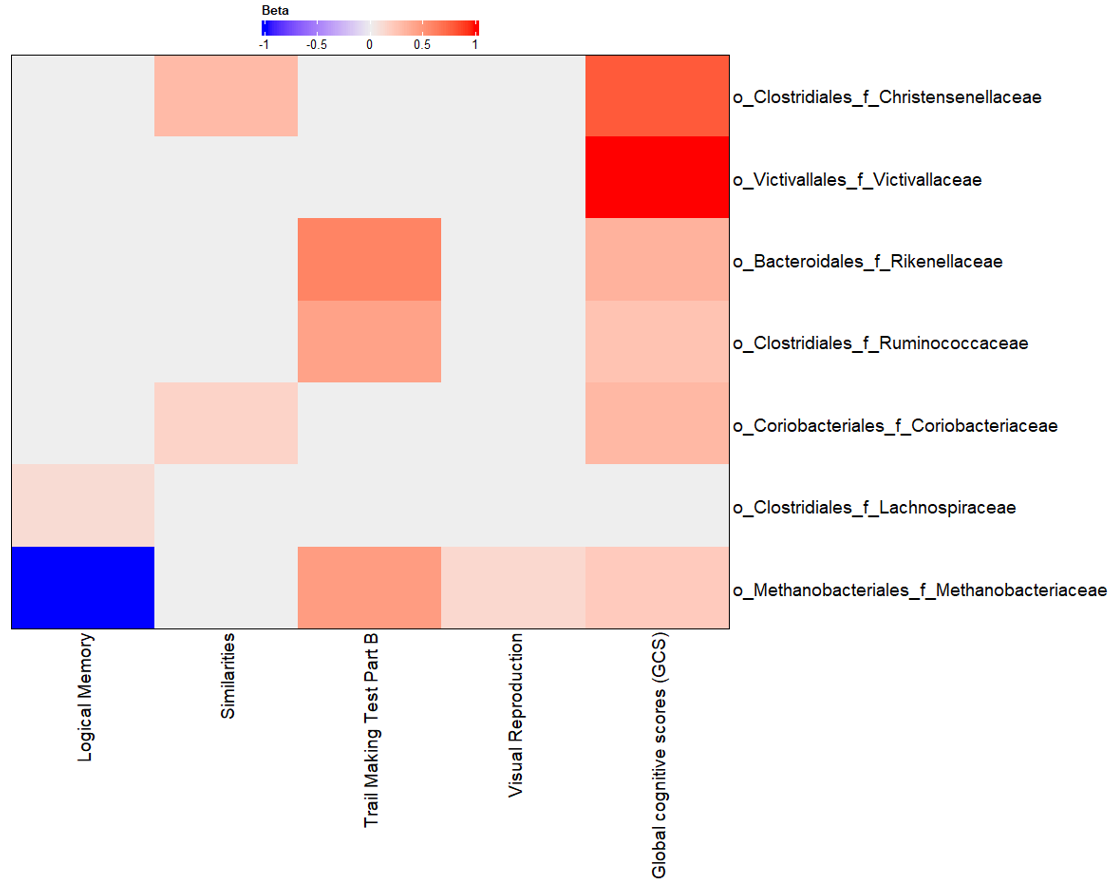


**Figure S11.** Multivariable association between gut microbiota and global cognitive scores. Heatmap depicting the significant (adjusted p-value < 0.05) taxa associated with GCS, along with scores of tests composing GCS at the family level.


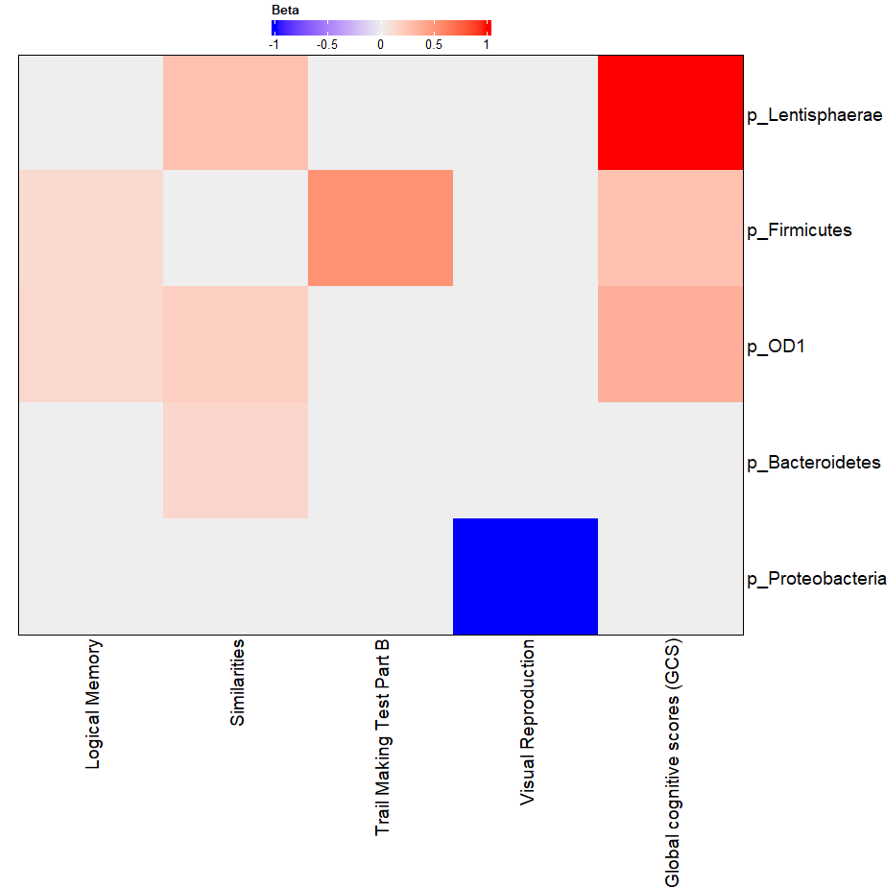


**Figure S12.** Multivariable association between gut microbiota and global cognitive scores. Heatmap depicting the significant (adjusted p-value < 0.05) taxa associated with GCS along with scores of tests composing GCS at the phylum level.

1. **Gut microbiome mediated the association of LE8 with global cognitive scores**


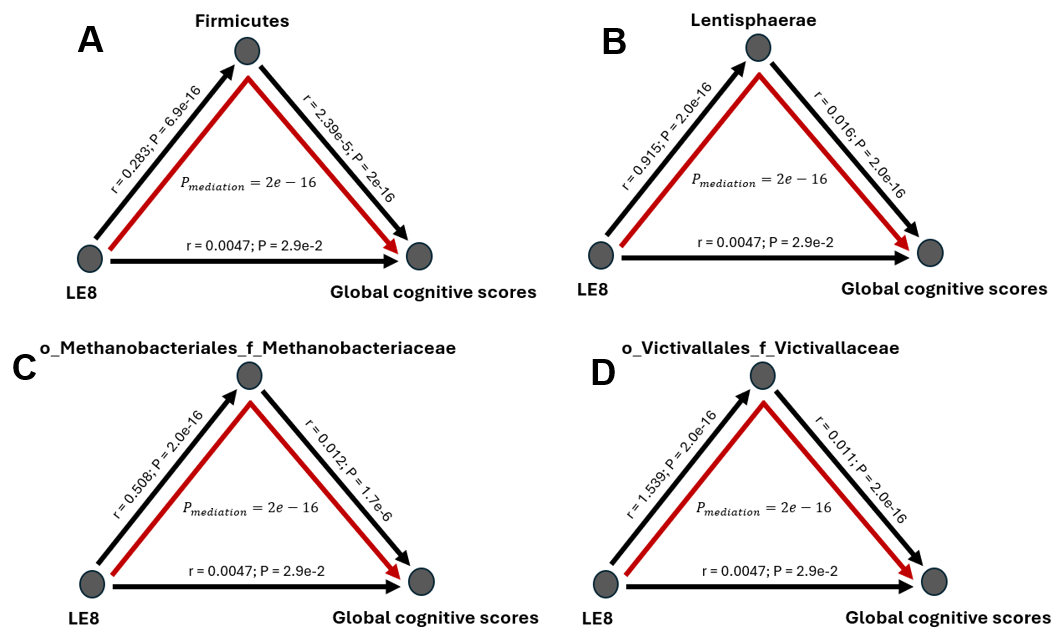


**Figure S13.** Mediation analysis of gut microbiome on the association between LE8 adherence scores and global cognitive scores. The mediation effect of bacteria *Firmicutes, Lentisphaerae, Victivallaceae,* and *Methanobacteriaceae* on the association between moderate LE8 adherence and lower cognition.
